# Structural Features of the Human Connectome That Facilitate the Switching of Brain Dynamics via Noradrenergic Neuromodulation

**DOI:** 10.3389/fncom.2021.687075

**Published:** 2021-07-14

**Authors:** Carlos Coronel-Oliveros, Samy Castro, Rodrigo Cofré, Patricio Orio

**Affiliations:** ^1^Instituto Milenio Centro Interdisciplinario de Neurociencia de Valparaíso, Universidad de Valparaíso, Valparaíso, Chile; ^2^Programa de Doctorado en Ciencias, Mención Biofísica y Biología Computacional, Universidad de Valparaíso, Valparaíso, Chile; ^3^Laboratoire de Neurosciences Cognitives et Adaptatives (LNCA), Faculté de Psychologie, Université de Strasbourg, Strasbourg, France; ^4^University of Strasbourg Institute for Advanced Studies (USIAS), Université de Strasbourg, Strasbourg, France; ^5^CIMFAV-Ingemat, Facultad de Ingeniería, Universidad de Valparaíso, Valparaíso, Chile; ^6^Institute of Neuroscience (NeuroPSI), Paris-Saclay University, Centre National de la Recherche Scientifique (CNRS), Gif-sur-Yvette, France; ^7^Facultad de Ciencias, Instituto de Neurociencias, Universidad de Valparaíso, Valparaíso, Chile

**Keywords:** whole brain model, neuromodulation, integration and segregation, network topology, noradrenaline, rich club organization

## Abstract

The structural connectivity of human brain allows the coexistence of segregated and integrated states of activity. Neuromodulatory systems facilitate the transition between these functional states and recent computational studies have shown how an interplay between the noradrenergic and cholinergic systems define these transitions. However, there is still much to be known about the interaction between the structural connectivity and the effect of neuromodulation, and to what extent the connectome facilitates dynamic transitions. In this work, we use a whole brain model, based on the Jasen and Rit equations plus a human structural connectivity matrix, to find out which structural features of the human connectome network define the optimal neuromodulatory effects. We simulated the effect of the noradrenergic system as changes in filter gain, and studied its effects related to the global-, local-, and meso-scale features of the connectome. At the global-scale, we found that the ability of the network of transiting through a variety of dynamical states is disrupted by randomization of the connection weights. By simulating neuromodulation of partial subsets of nodes, we found that transitions between integrated and segregated states are more easily achieved when targeting nodes with greater connection strengths—local feature—or belonging to the rich club—meso-scale feature. Overall, our findings clarify how the network spatial features, at different levels, interact with neuromodulation to facilitate the switching between segregated and integrated brain states and to sustain a richer brain dynamics.

## 1. Introduction

The human brain generates a rich repertoire of spatiotemporal dynamics characterized by the *integrated* and *segregated* functional states (Tononi, 2004). Information processed in parallel by domain-specific systems (segregated) is brought together (integrated) to guide adaptive behavior (Dehaene and Changeux, 2011). The balance between segregation and integration is essential to coordinate the local and global communication of neural information, it is needed to support a wide variety of cognitive functions, and has been proposed as a prominent organizational principle of the brain (Sporns, 2013; Cohen and D'Esposito, 2016; Shine, 2019; Wang et al., 2021). The dynamics and flexibility of brain activity, necessary for the coherent global functioning of the brain, enables the coexistence of segregated and integrated brain states (Kelso, 2012; Tognoli and Kelso, 2014; Wang et al., 2021).

Neuroimaging recording techniques such as electroencephalography (EEG) and functional magnetic resonance imaging (fMRI) allow the characterization of functional connectivity (FC) of the brain, from which the functional integration and segregation can be quantified using network theory tools (Bullmore and Sporns, 2009; González et al., 2016). The observed patterns of FC reflect the diversity of neuronal dynamics that emerge, among others, from the nonlinear dynamics of brain regions interconnected through structural connectivity (SC) (Deco and Jirsa, 2012; Lord et al., 2017; Guan et al., 2020). FC continuously evolves even in resting conditions (Allen et al., 2014; Hansen et al., 2015; Cabral et al., 2017), moreover, it changes across several tasks, highlighting the flexible nature of brain dynamics (Cohen and D'Esposito, 2016; Shine et al., 2016, 2019; Wang et al., 2021).

A plausible mechanism to facilitate—and regulate—transitions between different FC patterns are neuromodulatory systems. Neuromodulators do not directly excite neurons. Instead, they change their excitability and response to neurotransmitters, increasing or decreasing the probability of firing action potentials (Thiele and Bellgrove, 2018). The role of the cholinergic and noradrenergic systems in managing the segregation/integration balance has been evidenced in experimental (Shine et al., 2016, 2018b; Pfeffer et al., 2020), and theoretical frameworks (Shine et al., 2018a; Pfeffer et al., 2020; Coronel-Oliveros et al., 2021).

The noradrenergic system is involved in arousal when subjects engage in high-load cognitive tasks (Aston-Jones and Cohen, 2005; Shine et al., 2016, 2018b). For example, in fMRI recordings during an N-back task (for assessing working memory), the pupil diameter—a marker of noradrenergic tone (Reimer et al., 2016)—increases (Shine et al., 2016, 2018b). The principal source of noradrenaline in the cerebral cortex comes from the locus coeruleus (LC) (Fuxe et al., 2010). The GANE model of gain modulation (Mather et al., 2016; Lee et al., 2018), proposes that the noradrenergic system modulates neural response through an excitatory feedback loop between glutamate receptors on varicosities of LC projections and adrenergic β receptors on presynaptic glutamatergic neurons. At the same time, less activated neurons are suppressed through the action of adrenergic α_2_ autoreceptors expressed on the varicosities. The overall result comprises an increase of the neuron responsivity above a threshold, and a decrease of the responsivity below this threshold. This is equivalent to increasing the slope of the input-output sigmoid function, also named filter gain, as proposed in Servan-Schreiber et al. (1990) and Aston-Jones and Cohen (2005).

In a recent article (Shine, 2019), the noradrenergic system was considered to promote an integrated functional network configuration increasing the filter gain (Servan-Schreiber et al., 1990; Aston-Jones and Cohen, 2005; Mather et al., 2016; Thiele and Bellgrove, 2018). Computational studies (Shine et al., 2018a; Coronel-Oliveros et al., 2021) have also shown how the interplay between cholinergic and noradrenergic systems can regulate the segregation/integration balance. While recent theoretical articles point out that a non-uniform neuromodulation can explain better the effects of neuromodulatory systems in brain dynamics (Deco et al., 2018; Kringelbach et al., 2020), most studies so far have considered homogeneous neuromodulation, i.e., acting in all nodes in the same way.

There is evidence about the importance of network properties of the human connectome (Cabral et al., 2014; Zamora-López et al., 2016; Wang et al., 2019; Castro et al., 2020). For example, its hierarchical modular organization is needed to sustain a richer brain dynamics (Zamora-López et al., 2016; Wang et al., 2019). Then, the repertoire of network configurations, as a way to conceptualize the dynamical richness, can be affected by neuromodulation. Using a neural mass model to simulate neural activity, Shine et al. (2018a) showed that rich club regions were strongly neuromodulated compared with non-rich club members, especially between the transition from functional segregation to integration. This work notably suggests that some particular brain regions play a key role in the switching between different functional states via neuromodulation. Here, instead of quantifying what regions would be strongly neuromodulated, we studied how much the impact would be on integration and segregation when neuromodulating specific subsets of nodes, and analyzed the structural features that define the nodes that, upon modulation, have the largest effect on the network dynamics as a whole.

To investigate this issue, we built a whole-brain model based on the Jansen and Rit equations (Jansen et al., 1993; Jansen and Rit, 1995) coupled to a human SC matrix, that allows us to simulate the effect of the noradrenergic system on the functional integration and segregation features of the network (Coronel-Oliveros et al., 2021). The interaction between neuromodulation and structural connectivity was studied at three levels: at the global-scale, we used random surrogate connectomes that preserve the number and strength of connections but disrupt the global patterns. At the meso-scale, we determined whether the modulation of a node subset containing the anatomical rich club (Opsahl et al., 2008; Van Den Heuvel and Sporns, 2011) or the critical *s*-core (Garas et al., 2012; Eidsaa and Almaas, 2013), is optimal to produce a change in network dynamics, compared to randomly chosen subsets. At the local-scale, we explored which local properties define the set of nodes that, when being neuromodulated, maximize the effect on network dynamics.

We found that when we selectively neuromodulated the brain regions by the rich club (meso-scale property) or the high strength criteria (local-scale) the whole-brain network dynamics is most effectively modified. Additionally, we observed that surrogate connectomes reduced FC richness, compared with human SC, when neuromodulated. Overall, our findings clarify how the neuromodulation interacts with the anatomical network features at local-, meso-, and macro-scale levels in a whole-brain model to facilitate switching between segregated and integrated brain states.

## 2. Results

**Figure 1:**
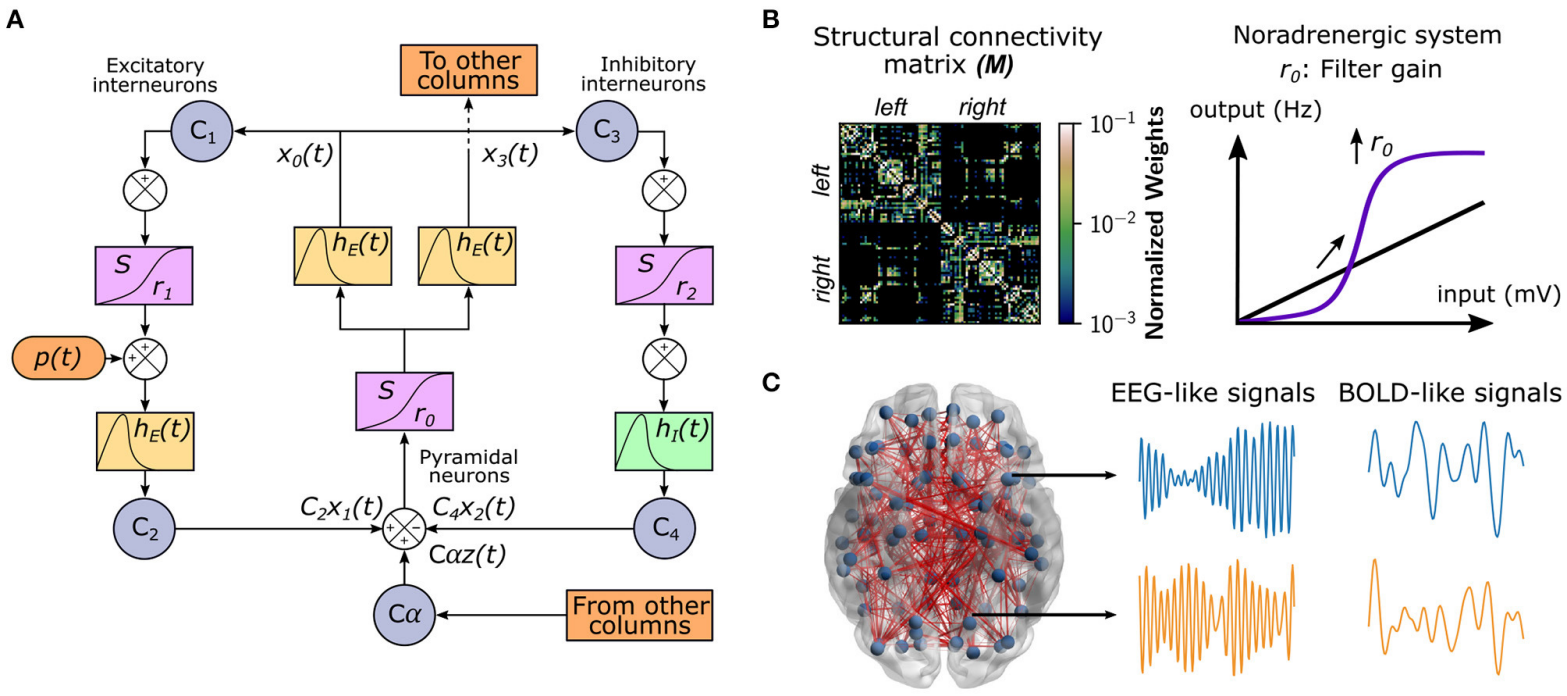
Whole-brain neural mass model with neuromodulation. **(A)** The Jansen and Rit model is composed of a population of pyramidal neurons with excitatory and inhibitory feedback mediated by interneurons. A series of constants *C*_*i*_ connect each population. The outputs are transformed from average pulse density to average postsynaptic membrane potential by an excitatory (inhibitory) impulse response function *h*_*E*_(*t*) [*h*_*I*_(*t*)]. Then, a sigmoid function *S* performs the inverse operation. Pyramidal neurons project to distant brain areas and receive both uncorrelated Gaussian-distributed inputs *p*(*t*) and inputs from other regions *z*(*t*), scaled by a global coupling parameter α. **(B)** Each node represents a cortical region, whose dynamics are ruled by the Jansen and Rit equations. The structural connectivity matrix, *M*, is the map of the connections (and their weights in the color bar) between cortical regions (row and columns of the matrix). The noradrenergic system increments pyramidal neuron responsivity to relevant stimuli with respect to noise, as a filter, by increasing the slope *r*_0_ of their sigmoid function. **(C)** The whole-brain model comprises 90 cortical and subcortical regions linked by a human connectome. For each region, the model produces both EEG-like and BOLD-like signals. The brain figure was obtained using the BrainNet Viewer (Xia et al., 2013).

To study the effect of neuromodulatory systems on the integrative/segregative capacities of the human connectome, we used a whole-brain model of brain activity (Coronel-Oliveros et al., 2021). In this model, each node corresponds to a brain area represented by a neural mass, which consists of three populations (Jansen et al., 1993; Jansen and Rit, 1995): pyramidal neurons, excitatory, and inhibitory interneurons (Figure 1A). We used the same parameters as in Jansen et al. (1993) and Jansen and Rit (1995), except the connectivity constant from inhibitory interneurons to pyramidal neurons *C*_4_, which we modified to *C*_4_ = 0.5*C*, being *C* the original intra-area connectivity constant of the model. The nodes are connected through a weighted undirected structural connectivity matrix derived from human MRI data (Deco et al., 2018), parcelated in 90 cortical and sub-cortical regions with the automated anatomical labeling (AAL) atlas (Tzourio-Mazoyer et al., 2002; Figure 1B). Pyramidal neurons connect regions (or nodes) because it is considered that long-range projections are mainly excitatory (Gilbert et al., 1990; McGuire et al., 1991). The simulations generate firing rates in each node of the network, which was used as an input to a generalized hemodynamic model (Stephan et al., 2007). In this way, we obtained fMRI BOLD-like signals (Figure 1C) from which we built the FC matrices.

We modeled the influence of the noradrenergic system through the manipulation of the filter gain (Aston-Jones and Cohen, 2005; Shine, 2019; Figure 1B). The filter gain *r*_0_ modifies the sigmoid function slope of pyramidal neurons, increasing their responsivity to relevant stimuli, decreasing the response to low amplitude stimuli, and boosting the signal-to-noise ratio.

### 2.1. Human Structural Connectivity Enhances Dynamical Richness

**Figure 2:**
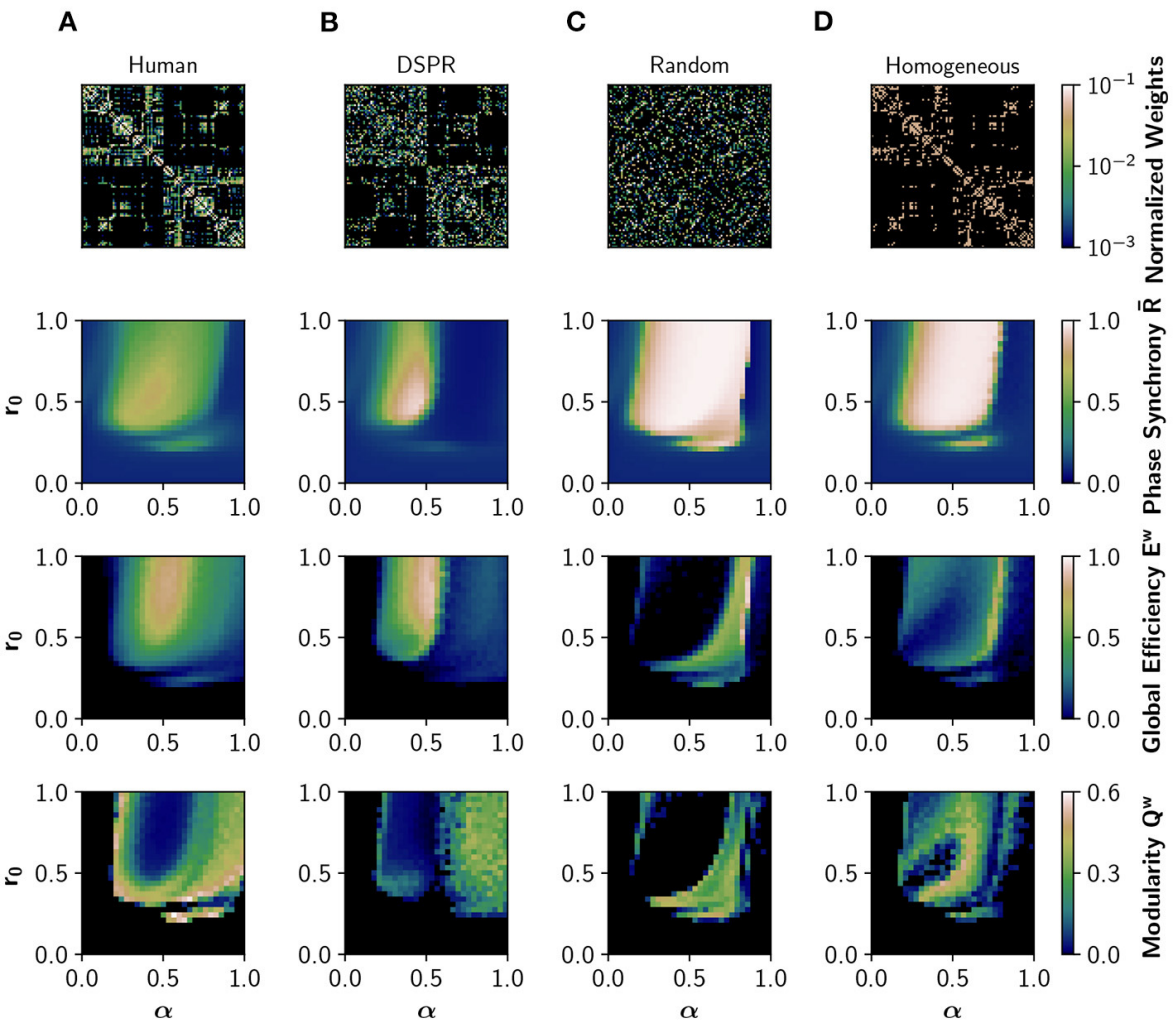
Effects of network structure in neural synchronization and integration. From top to bottom: structural connectivity matrix, phase synchrony $\overset{-}{R}$, global efficiency *E*^*w*^ (a measure of integration), and modularity *Q*^*w*^ (measure of segregation), obtained in the model with different structural connectivities. $\overset{-}{R}$ was obtained from EEG-like simulated activity, while *E*^*w*^ and *Q*^*w*^ were calculated using the FC obtained from the corresponding fMRI BOLD-like traces. **(A)** Human structural connectivity (Human). **(B)** Degree- and strength-preserving randomized version of Human (DSPR). **(C)** Randomized version of Human, where weight values were shuffled across the full matrix (Random). **(D)** Homogeneous version of Human, having the same weight in all connections (Homogeneous).

First, we analyzed how neuromodulation depends on the connectivity pattern of the human connectome by using different randomized surrogate connectomes. We employed a degree- and strength-preserving randomization (DSPR), which randomizes the structural connectivity while preserving original degree and strength distributions (Figure 2B); in this way we can study the effect of disrupting the global connectivity without altering the local nodal properties. In addition, we employed a complete randomization of the structural connectivity (Figure 2C), which does not preserve the degree and strength distributions. Finally, a homogenization (binarization) of the connectome was considered (Figure 2D); this surrogate preserves the topology, disrupting the non-uniform weight distribution. We simulated EEG-like and fMRI BOLD-like signals from the Jansen and Rit model at different combinations of α ∈ [0, 1] and *r*_0_ ∈ [0, 1] parameters. Here, the value of the parameters is equal for all the nodes, and we refer to this case as uniform neuromodulation. We computed the mean of the Kuramoto order parameter, also known as phase synchrony $\overset{-}{R}$, the global efficiency, *E*^*w*^, and the modularity, *Q*^*w*^, as measures of global phase synchronization, integration, and segregation, respectively. Global efficiency is a measure of integration defined as the inverse of the characteristic path length (Rubinov and Sporns, 2010). High values of *E*^*w*^ represent an efficient coordination between all pairs of nodes in the network, a signature of integration. Modularity is a measure of segregation based on the detection of network communities, or modules (Rubinov and Sporns, 2010); high modularity *Q*^*w*^ is associated with segregation and vice-versa.

Figure 2A shows how neuromodulation of the human connectome causes a shift of the model toward a synchronized and integrated state, with maximum integration observed in an intermediate region of the parameter space, as previously reported in Shine et al. (2018a) and Coronel-Oliveros et al. (2021). The synchrony $\overset{-}{R}$ has an upper bound of 0.76, that is, the network never fully synchronizes. The transition is gradual, with many regions showing an intermediate behavior characterized by higher metastability and richer dynamics (Zamora-López et al., 2016; Shine et al., 2018a; Coronel-Oliveros et al., 2021). Moreover, the region of the parameter space where $\overset{-}{R}$ increases matches the increment in global efficiency, *E*^*w*^, verifying a link between the fast dynamics of EEG and the slower one of fMRI-BOLD.

We repeated the same exploration using the DSPR surrogate connectome (Figure 2B; Rubinov and Sporns, 2011). The area of synchronized activity in the parameter space (*r*_0_, α) is reduced, and a spot of over-synchronized activity can be appreciated. Most importantly, the area of intermediate values of synchrony and integration is largely reduced, suggesting a reduction of dynamical richness. When the connectivity matrix is completely randomized (Figure 2C), or made homogeneous by assigning equal weights to all connections (Figure 2D), neuromodulation produces a large area of over-synchronized activity in the parameter space and fewer regions with intermediate behavior.

The dramatic decrease in *E*^*w*^ in Figures 2C,D is a consequence of the over-synchronization ($\overset{-}{R}\approx 1$) triggered by randomization. When signals are highly synchronized in our model, the envelope in the alpha band of the EEG (between 8 and 13 Hz) becomes flat, and so does the BOLD-like signal calculated with the hemodynamic model (Foster et al., 2016). For this reason, this drop in *E*^*w*^ should not be interpreted as a reduction of integration but a limitation of the hemodynamic model we employed in input simulations. Nevertheless, an over-synchronized regime of activity is a feature never found in the healthy brain (Miron-Shahar et al., 2019).

Thus, in line with several previous reports (Cabral et al., 2014; Zamora-López et al., 2016; Wang et al., 2019; Fukushima and Sporns, 2020), disrupting the organization of the human connectome (or the weight relationships between nodes) causes over-synchronization, and highly metastable regimes can not be easily reached employing neuromodulation.

In the following, we will study which local- or meso-scale organization features are determinant in the effect of neuromodulation of human connectome by evaluating the network behavior when changing the *r*_0_ parameter in subsets of network nodes.

### 2.2. Neuromodulation of High-Strength Nodes Promotes Better Functional Integration

In this section, we investigate the impact on functional integration when an increasing number of nodes are neuromodulated. The order in which nodes are modulated is defined considering nodal measures obtained from the structural matrix *M*. We calculated, for each node *i* ∈ [1…*n*]: node strength, $K_{i}^{w}$, nodal efficiency, $E_{i}^{w}$, and clustering coefficient, $C_{i}^{w}$ (Rubinov and Sporns, 2010). The superscript *w* indicates the use of the weighted versions of the measures. Then, for each metric, nodes were ordered either from high to low or from low to high. We fixed the global coupling α = 0.65, and swept *r*_0_ ∈ [0.33, 1] and the number of nodes being neuromodulated in [0, 90] in steps of three. As before, we used the EEG-like and BOLD-like signals to extract synchrony, integration, and segregation.

**Figure 3:**
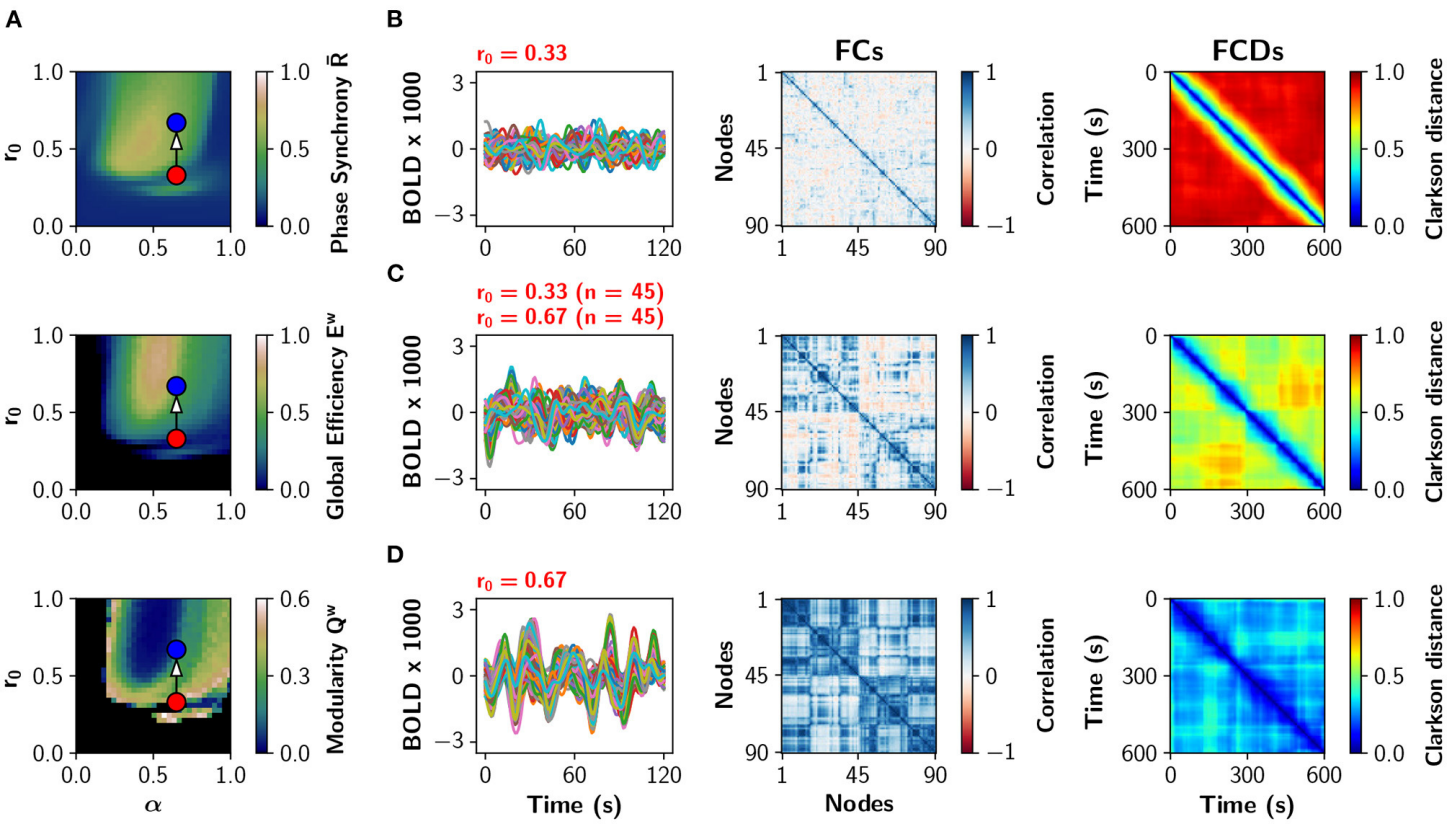
Partial noradrenergic neuromodulation. **(A)** The (α, *r*_0_) parameter space showing phase synchrony $\overset{-}{R}$, global efficiency *E*^*w*^, and modularity *Q*^*w*^, for the Human connectome (same as in Figure 2A). Blue and red dots are references for **(B–D)**. **(B–D)** BOLD-like signals, FC and FCD matrices obtained when all nodes have α = 0.65 and **(B)** *r*_0_ = 0.33; **(C)** 45 nodes have *r*_0_ = 0.33 and 45 nodes have *r*_0_ = 0.67; and **(D)** all nodes have *r*_0_ = 0.67. In **(C)**, the 45 nodes with the highest strength were modified to *r*_0_ = 0.67.

A particular example of partial neuromodulation is shown with some detail in Figure 3. The (α, *r*_0_) parameter space is shown in Figure 3A depicting global phase synchrony $\overset{-}{R}$, global efficiency *E*^*w*^ and modularity *Q*^*w*^ in a uniform neuromodulation scenario (all nodes identical). Figure 3B shows sample BOLD traces, the functional connectivity (FC) and the functional connectivity dynamics (FCD) matrices for α = 0.65, *r*_0_ = 0.33 (red dot in Figure 3A). The FCD matrix visually represents the dynamical richness of the network, by computing time-dependent FCs using sliding windows (Cabral et al., 2017; Orio et al., 2018). Then, FCs are vectorized and compared to each other using the Clarkson distance (Clarkson, 1936), resulting in a matrix of time vs. time. At the bottom, Figure 3D shows the same analysis for α = 0.65, *r*_0_ = 0.67 (blue dot in Figure 3A). In the middle, Figure 3C shows the results when only half of the nodes have been neuromodulated to *r*_0_ = 0.67 while the rest remain with *r*_0_ = 0.33. As the number of nodes with *r*_0_ = 0.67 increases, the FC matrices become more integrated (high *E*^*w*^ and low *Q*^*w*^ values). Similarly, the FCD matrices change from incoherence (red FCD), to exhibit multi-stable behavior (FCD with yellow-green patches), and finally to show correlated FC patterns (blue FCD). In summary, the increment of the number of neuromodulated nodes increases phase synchrony, functional integration, and the time correlation of FCs captured by the FCD.

**Figure 4:**
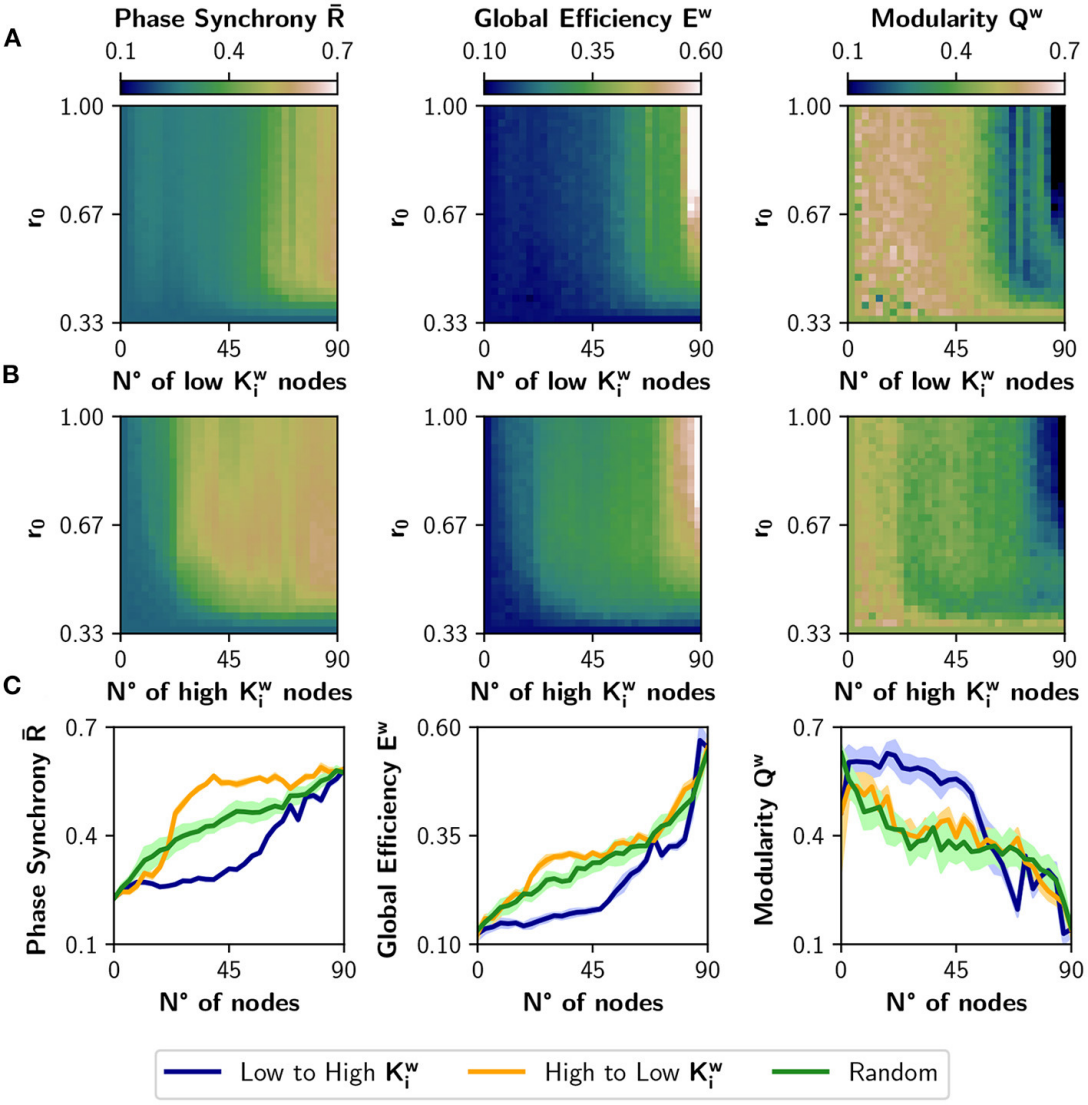
Incremental neuromodulation based on node strength. **(A)** Effect of the neuromodulation on phase synchrony $\overset{-}{R}$, global efficiency *E*^*w*^, and modularity *Q*^*w*^, at different combinations of *r*_0_ and number of neuromodulated nodes. Nodes were affected by neuromodulation according to their strength, from low to high. **(B)** Effect of the neuromodulation of nodes sorted from high to low strength. **(C)** Metrics as a function of the number of neuromodulated nodes, for *r*_0_ = 0.67 as target value. Blue curves for neuromodulation of nodes with low strength, orange the opposite, and green for a random ordering of the nodes. Shaded areas correspond to 95% confidence intervals, for 10 realizations.

Figure 4 shows the result of neuromodulating *r*_0_ with a target value in the [0.33, 1] interval and with the number of neuromodulated nodes ranging from 0 to 90. The order in which nodes are neuromodulated is either from low to high $K_{i}^{w}$ (Figure 4A) or viceversa (Figure 4B). When the number of neuromodulated nodes is large, $\overset{-}{R}$ and *E*^*w*^ raise markedly in both cases; the opposite can be observed for *Q*^*w*^. However, picking the nodes of high strength first (Figure 4B) has greater impact in the change of those metrics. The difference is best appreciated in Figure 4C, where we selected a target *r*_0_ value of 0.67. There, the curves for the high to low $K_{i}^{w}$ sorting (in orange) present a larger effect at the beginning, compared with the low to high $K_{i}^{w}$ sorting (in blue). We can conclude that nodes with higher strength have a greater impact on functional integration, and inspection of the colormaps of Figures 4A,B reveals that this is true for almost all values of target *r*_0_. The results were also compared with a random selection of nodes for neuromodulation (green curves). As the blue curve is mainly below the green curve, neuromodulation of nodes with low $K_{i}^{w}$ produces less synchronized and integrated dynamics than expected by a random neuromodulation. In contrast, choosing high $K_{i}^{w}$ nodes is not different from random selection, when looking at the measures of integration and segregation. In consequence, there is a range (or possibly a set) of nodes that produce a robust integration when neuromodulated, compared to a random choice of nodes.

**Figure 5:**
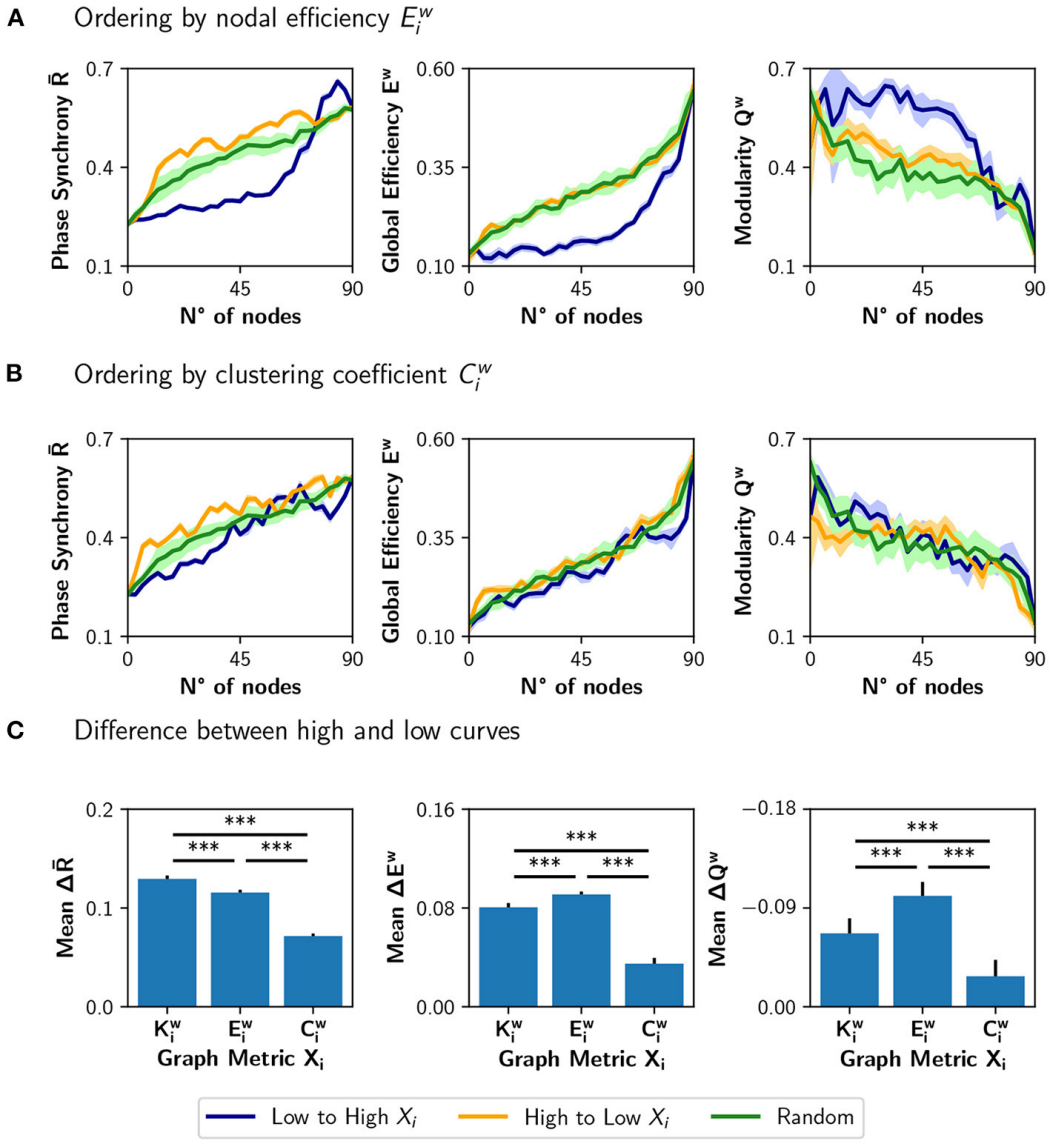
Incremental neuromodulation based on nodal efficiency and clustering coefficient. **(A,B)** Phase synchrony $\overset{-}{R}$, global efficiency *E*^*w*^, and modularity *Q*^*w*^ as a function of the number of neuromodulated nodes, for *r*_0_ = 0.67 as target value. In **(A)**, nodes were sorted according to nodal efficiency, $E_{i}^{w}$, and in **(B)** according to the clustering coefficient, $C_{i}^{w}$. Blue curves for sorting of nodes from low to high (of the particular metric), orange is from high to low, and green for a random sorting of the nodes. **(C)** Difference between the area under the curve (AUC) of high vs. low sorting, averaged over the 10 realizations. Shaded areas in **(A,B)** correspond to 95% confidence intervals, and barplots were built using the mean ± standard deviation. ***p < 0.001.

We compared the results of sorting the nodes based on strength $K_{i}^{w}$, with ranking the nodes based on nodal efficiency $E_{i}^{w}$ or clustering coefficient $C_{i}^{w}$ (Figure 5). A node with a high $E_{i}^{w}$ has many short paths to the rest of the nodes of the network, while a high $C_{i}^{w}$ is expected for nodes whose neighbors are also connected between them. Figure 5A shows the result of modulating an increasing number of nodes from a basal *r*_0_ = 0.33 to a target *r*_0_ = 0.67, when the nodes are ordered from low to high or high to low $E_{i}^{w}$. The results are similar to the ones obtained using the strength $K_{i}^{w}$: neuromodulation of nodes of high $E_{i}^{w}$ has a greater impact in synchronization and integration, compared with the nodes of low $E_{i}^{w}$. Here, the random sorting of nodes is similar to the high to low $E_{i}^{w}$. However, when the nodes are sorted according to their clustering coefficient $C_{i}^{w}$ (Figure 5B), there is little difference compared to random sorting.

When comparing the results in Figures 5A,B with the neuromodulation of a random subset of nodes (green curves), there is no clear advantage of selecting the nodes with high $E_{i}^{w}$ or $C_{i}^{w}$. Despite the increase in $\overset{-}{R}$ being slightly higher for the orange curves, compared with the green curves, the difference in *E*^*w*^ is unnoticeable, except in *Q*^*w*^ when ordering the nodes from high to low $E_{i}^{w}$. These results contrast with the ones in Figure 4C, where the neuromodulation of nodes with high $K_{i}^{w}$ produced an increase in synchronization and integration higher than random neuromodulation.

To summarize these results, we computed the difference between the area under the curve (AUC) for the high-to-low minus low-to-high (orange minus blue AUCs; Figure 5C). A larger difference implies a higher impact of neuromodulating first the nodes with a higher value of the chosen metric in synchronization, integration, and segregation. The mean difference in the AUCs for the measures $\Delta \overset{-}{R}$, Δ*E*^*w*^, and −Δ*Q*^*w*^ (note that the sign is inverted for visualization purposes), is lower for $C_{i}^{w}$ than for $K_{i}^{w}$ and $E_{i}^{w}$ (*p* < 0.001 for all comparisons using Student's *t*-test).

### 2.3. Neuromodulation of Rich Club Nodes Strongly Impacts Functional Integration

**Figure 6:**
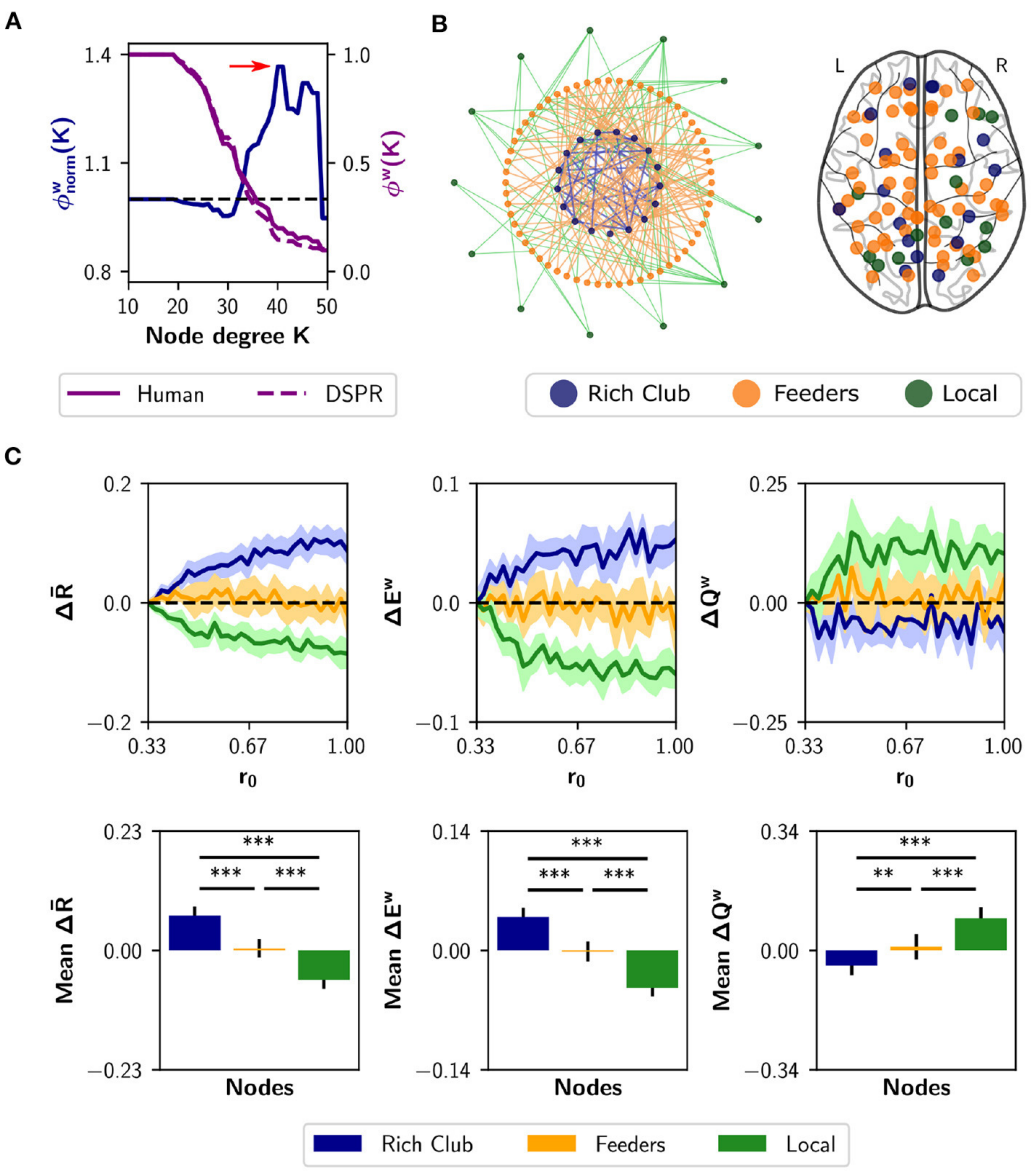
Neuromodulation based on the rich club organization. **(A)** Normalized and weighted rich club coefficient $\phi_{norm}^{w}\left(K\right)$ (blue curve), as a function of the degree-based threshold *K*. This coefficient was calculated as the ratio between the human rich club coefficient (purple solid curve) and the mean coefficient for random surrogates (DSPR, purple dashed curve). The red arrow marks the point in which $\phi_{norm}^{w}\left(K\right)$ is maximal; rich club nodes were found at that point. **(B)** Schematic depiction of the rich club, feeders (not belonging to the rich club, but connected with it) and local nodes, (connected only to feeders). At the right, we show a glass brain with the nodes identified as rich club (*n* = 17 nodes), feeders (*n* = 60), and local (*n* = 13). **(C)** Changes in synchrony $\overset{-}{R}$, global efficiency *E*^*w*^ and modularity *Q*^*w*^ when neuromodulating 24-node sets containing the rich club (blue), local nodes (green), or only feeders (orange). The results are shown as the difference with respect to a random subset of nodes of equal size (null case). The bottom row summarizes the area under the curve (AUC) for each metric and nodal category, averaged over the 10 realizations. Shaded areas correspond to 95% confidence intervals, and bar plots were built using the mean ± standard deviation. **p < 0.01, ***p < 0.001.

Node strength, nodal efficiency and clustering coefficient are considered local-scale properties, i.e., they belong to each node. Several meso-scale network properties have been described as being determinant for network dynamic too, such as the rich club organization (Van Den Heuvel and Sporns, 2011) and the *s*-core (Hagmann et al., 2008; Garas et al., 2012; Eidsaa and Almaas, 2013; Castro et al., 2020). We identified the nodes belonging to the “rich club,” using the weighted rich club coefficient ϕ^*w*^(*K*), where *K* is a threshold based on degree (Opsahl et al., 2008). The rich club comprises a subset of the graph, thresholded at *K*, in which nodes are more strongly interconnected than expected by chance (Van Den Heuvel and Sporns, 2011). The coefficient is normalized using random surrogates $\phi_{rand}^{w}\left(K\right)$, in our case DSPR surrogates (Rubinov and Sporns, 2011). If the normalized coefficient $\phi_{norm}^{w}\left(K\right)$ is greater than 1, the network has a rich club organization at threshold *K*. Figure 6A shows a plot of $\phi_{norm}^{w}\left(K\right)$ (blue) as a function of *K*. The red arrow marks the point in which the normalized coefficient is maximal [$\phi_{norm}^{w}\left(K\right)=1.367,p<0.002$]. Then, we identified feeder nodes—nodes that do not belong to the rich club but are connected to at least one of its members—and local nodes—connected to feeders but not to the rich club (Figure 6B). We found 17 nodes belonging to the rich club, 60 feeders and 13 local nodes (Figure 6B). The rich club members are the brain regions in Table 1.

**Table 1:** List of regions belonging (X) to the rich club, the *S*_3_ category, and the 17 nodes with highest strength.

**Brain regions**	**Rich club**	***S*_3_ core**	**Top strength**
Posterior cingulate gyrus (L, R)	X	X	X
Precuneus (L, R)	X	X	X
Calcarine fissure (L, R)		X	X
Cuncus (L, R)		X	
Cuneus (L, R)		X	X
Caudate nucleus (R)	X		
Hippocampus (L, R)	X		
Insula (L)			X
Middle occipital gyrus (L)	X		X
Pallidum (L, R)	X		
Putamen (L, R)	X		X
Rolandic Operculum (L)			X
Superior dorsal gyrus, dorsolateral (L, R)	X		
Superior frontal gyrus, orbital (L)			X
Superior occipital gyrus (L, R)			X
Superior frontal gyrus, medial (L)	X		X
Thalamus (L, R)	X		

**Figure 7:**
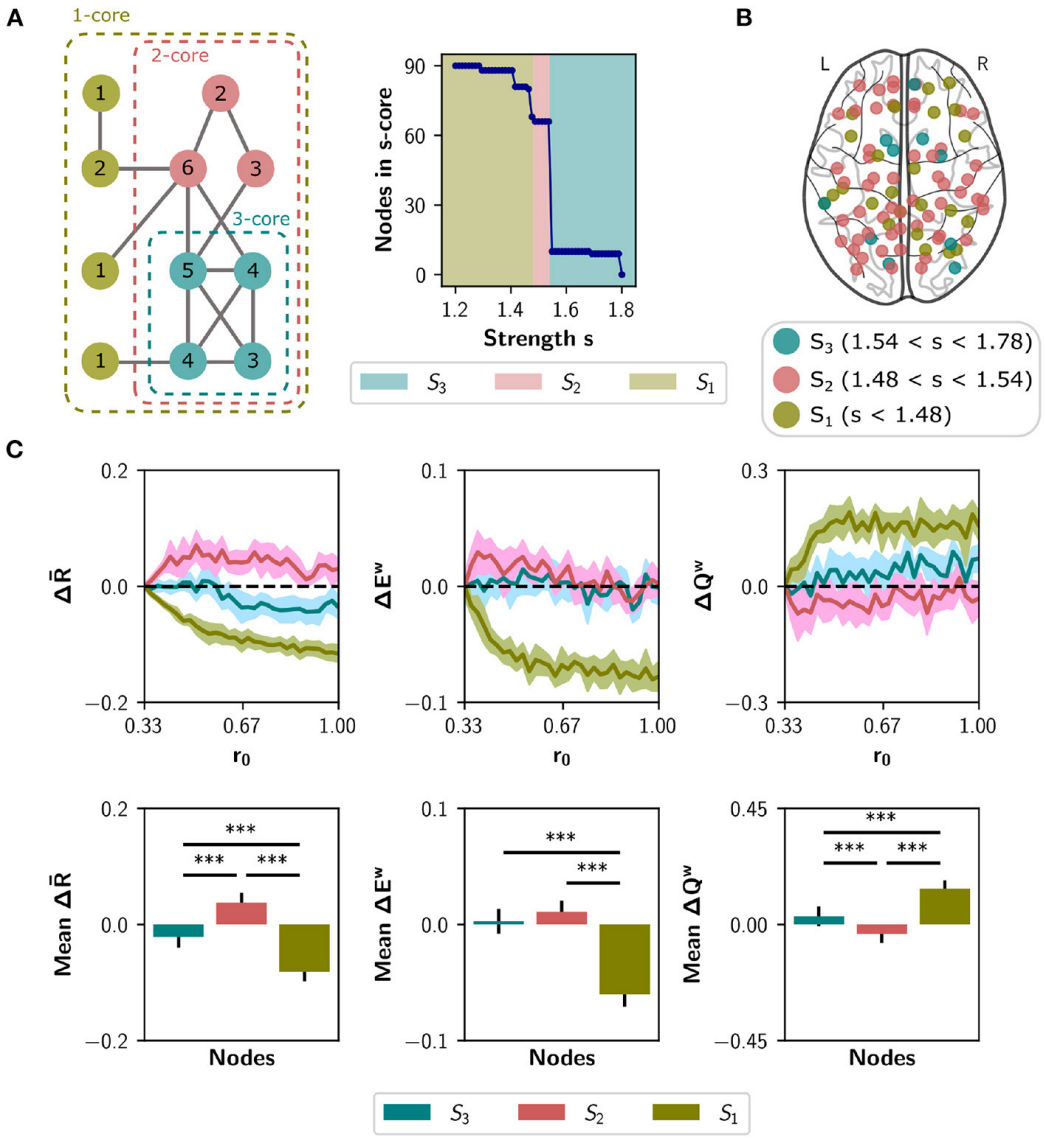
Neuromodulation based on the *s*-core decomposition. **(A)** *s*-core decomposition. At the left, example based on degree (*k*-core). Nodes are recursively removed based on a degree threshold. The remaining nodes form a subgraph or core where all nodes have a within-degree above the threshold. For example, the three-core of the figure corresponds to a subgraph where all nodes have a degree of three or more. The numbers on the circles correspond to nodes' degree. The right plot shows the number of remaining nodes in *s*-core after applying the strength-based threshold *s*. **(B)** Brain regions identified using the *s*-core decomposition. We defined three categories, considering a range of *s* values: *S*_3_ (*n* = 10 nodes), *S*_2_ (*n* = 56), and *S*_1_ (*n* = 24). **(C)** Changes in synchrony $\overset{-}{R}$, global efficiency *E*^*w*^ and modularity *Q*^*w*^ when neuromodulating 24-node sets containing the *S*_3_ nodes (blue), *S*_1_ nodes (olive green), or only *S*_2_ nodes (pink). *S*_1_ and *S*_3_ sets were complemented with random nodes from *S*_2_ to obtain sets of 24. Results are shown as the difference with respect to a random subset of nodes of equal size (null case). The bottom row summarizes the area under the curve (AUC) for each metric and nodal category, averaged over the 10 random seeds. Shaded areas correspond to 95% confidence intervals, and bar plots were built using the mean ± standard deviation. ***p < 0.001.

*L, left hemisphere; R, right hemisphere. The regions listed here are the same displayed in the glass brains of Figures 6, 7*.

As the analysis of the rich-club properties of the human SC defines sub-networks, instead of sorting the nodes, we chose a different approach than the neuromodulation of increasing subsets of nodes. Here, we simulated neuromodulation of a fixed-size subset of nodes, that included all nodes belonging to a certain category (rich club, feeders, or local). Because the categories differ in size, we complemented the rich club and local nodes with 7 and 11 nodes, respectively, selected randomly from the feeders. For the last one, we randomly chose 24 feeder nodes. Also, we had a null case, composed of 24 nodes randomly selected from the complete set of nodes. We repeated the random selection of nodes with 10 realizations, always using subsets of 24 nodes. The nodes started with a basal *r*_0_ value of 0.33, and *r*_0_ was swept up to 1 but only in the designated subset of nodes. For each *r*_0_ increment, we measured $\overset{-}{R}$, *E*^*w*^, and *Q*^*w*^. Then, we subtracted to each measurement the result of the null case. The results are shown in Figure 6C. Neuromodulation of the rich club nodes produces an increase in synchronization and integration, and a decrease in modularity, above chance. The difference becomes more pronounced with further increments of *r*_0_. Opposite results were observed for the subsets containing local nodes. Finally, neuromodulation of subsets containing only feeder nodes produce no difference compared to random selection of nodes. As a summary index, we calculated the AUC for each nodal category (Figure 6C). Considering the three measurements, the AUC is higher (lower in the case of modularity *Q*^*w*^) for the rich club respect to feeders and local, and higher (lower in the case of *Q*^*w*^) between feeders and local (*p* < 0.001 for all comparisons using Student's *t*-test). Our results show that noradrenergic neuromodulation of a subset including the rich club nodes has a greater impact on integration compared to the feeders, locals, and a random selection of nodes.

As previously shown, functional integration is also achieved by neuromodulation of highest strength nodes. To highlight the difference between the local and meso-scale approaches, we quantified the overlap between the rich club nodes and the 17 nodes with higher strength. We found that only 8 members of the rich club belong to the subset of 17 nodes with higher strength (Table 1). Thus, there are some high-strength key nodes that do not belong to the rich club, that promote functional integration via neuromodulation.

To explore a second meso-scale network organization, we performed a *s*-core decomposition (Garas et al., 2012; Eidsaa and Almaas, 2013) that classifies nodes according to their core-periphery organization (Hagmann et al., 2008; Figure 7A). We defined three categories considering a range of critical *s*-core values: *S*_3_ with 10 nodes (1.54 < *s* < 1.78), *S*_2_ with 56 nodes (1.48 < *s* < 1.54), and *S*_1_ with 24 nodes (*s* < 1.48; Figure 7B). The critical *s*-core is defined as the maximal value of *s* at which nodes are still connected to the network. Thus, *S*_3_ are nodes connected within them with highest strength, *S*_2_ middle-strength nodes, and *S*_1_ the nodes with the lowest strength. The *S*_3_ subset comprises the brain regions shown in Table 1.

We simulated the neuromodulation in subsets of 24 nodes, containing either the *S*_3_ or the *S*_1_ category, and complementing *S*_3_ with 14 random nodes from *S*_2_ as done with the rich club. A third group was built with 24 nodes randomly selected from *S*_2_, and all groups were compared to a random selection of 24 nodes from the whole set. As shown in Figure 7C, the selection of *S*_2_ nodes for neuromodulation shows the largest effect in $\Delta \overset{-}{R}$, Δ*E*^*w*^, and Δ*Q*^*w*^, compared with *S*_1_ nodes (*p* < 0.001 using Student's *t*-test) and compared to the selection of *S*_3_ nodes (*p* < 0.001, except for Δ*E*^*w*^ with *p* = 0.106). Thus, nodes belonging to the highest *s*-core (nodes of the highest within-strength sub-network) do not behave like the rich club, as their neuromodulation does not have the highest impact on network synchronization and integration/segregation properties.

## 3. Discussion

In this work, we sought to identify the relationship between structural features of the human connectome and the specific set of regions that, when neuromodulated in a biologically realistic whole-brain model, produce a significant increase in functional integration. We found that the global organization of the connectome sustains rich metastable and partially synchronized states, essential to the effects related to neuromodulation. At the meso- and local-scales, nodes belonging to the anatomical rich club, and those having high nodal strength, produce a marked increase in functional integration (and a decrease in segregation) when neuromodulated.

Our results show that the whole-brain model exhibits over-synchronized behavior when using surrogate connectomes, restricting the dynamic features of the model. This result is in the same line as other previous findings (Cabral et al., 2014; Zamora-López et al., 2016; Wang et al., 2019; Fukushima and Sporns, 2020). Here, we show this behavior in the (α, *r*_0_) parameter space, where simulations with randomized connectomes show either incoherent or over-synchronized activity. Using a whole-brain model to simulate and fit magnetoencephalography (MEG) resting-state recordings, Cabral et al. (2014) found not only that randomized and homogenized versions of the human structural connectivity did not fit empirical data; moreover, they found that the fit was maximal in the metastable region of the parameter space, when unsynchronized (segregated) and synchronized (integrated) regimes of activity coexist. In the same way, Fukushima and Sporns (2020) using more complex surrogate data in the context of whole-brain models, found features of the human connectome that better capture the dynamic fluctuations in fMRI resting-state recordings. Additionally, computational studies conducted by Zamora-López et al. (2016) showed that the human connectome better maximizes functional complexity in fMRI recordings, compared with different surrogate connectomes. Finally, Wang et al. (2019) analyzed how the hierarchical modular structure of the human connectome enables the coexistence of segregated and integrated functional states, also with the use of network surrogates in which hierarchical levels were controlled. Our study interpret the explorations of the parameter space as levels of neuromodulation, that allow the brain to tune its integration or segregation levels to environmental demands. However, neuromodulation cannot bring back a dynamically rich regime to a network without a structural connectome that sustains it.

At the local level, the effects of neuromodulation strongly depend on the characteristics of the nodes in the human connectome. In our model, the nodes with high strength are the ones that better facilitate functional integration when neuromodulated. This result resonates with a recent work by Herzog et al. (2020), who studied a whole-brain model fitted to reproduce the effects of lysergic acid diethylamide (LSD) in resting-state brain dynamics. In their model, the serotonergic-induced changes in nodal entropy correlated positively with node strength. Notably, the correlation disappears when the human connectome was randomized without preserving the strength distribution, emphasizing the importance of the specific organization of the human connectome in shaping brain dynamics. Interestingly, the entropy changes described by Herzog et al. (2020) are poorly explained by the 5*HT*_2*A*_ receptor density map, obtained by PET (Beliveau et al., 2017), and depends on both node strength and receptor density. Thus, the interaction between the structural connectivity, receptor density, and neuromodulation is not straightforward. A similar complex picture arises when our results are contrasted with receptor maps of noradrenergic receptors (see below).

Network hubs, or nodes belonging to the rich club or network's ignition core, can be critical elements for binding information of segregated brain regions, that is, to integrate information across brain areas (Griffa and Van den Heuvel, 2018; Castro et al., 2020). Considering the relevance of integration for the brain function (Tononi, 2004), and the noradrenergic influence on integration (Shine, 2019), we hypothesized that anatomical network hubs are pivotal elements for promoting functional network integration. Our results confirmed this hypothesis, being the neuromodulation of rich club nodes the one that most effectively facilitates functional integration and synchronization of brain activity. This result agrees with findings reported in a fMRI resting-state model of the brain by Deco et al. (2017), where removing the rich club nodes causes a larger decrease in integration compared to the removal of the non-rich club members. Similar results have been found in computational models of noradrenergic neuromodulation where rich club nodes are strongly neuromodulated causing functional networks to switch from segregation to integration (Shine et al., 2018a).

Notably, neuromodulation of nodes belonging to the critical *s*-core (the maximally inter-connected core) does not promote integration as the rich club nodes do. Both meso-scale analyses rely on sets of nodes organized with strong connection weights. However, they do it differently. The rich club coefficient threshold is based on degree, and rich club members are highly connected between them as well as with the non-rich club members. In contrast, the *s*-core decomposition find subsets of nodes highly interconnected at strength *s*, but not necessarily well connected to the rest of the network. Thus, the whole-network changes are more easily achieved if the set of nodes to be neuromodulated is highly connected both between them and with the rest of the network. The rich-club organization captures additional information that is missing in the local (weight) analysis. For example, the 17 rich club nodes have an overlap of ≈50% with the 17 highest strength nodes. In contrast, nodes belonging to the *S*_3_ category are the nodes of the highest strength in the network; however, they cannot boost functional integration to the same extent as the rich club nodes.

Part of the brain regions we found in the rich club support high order brain functions. For example, frontoparietal regions play an important role in cognition, and are markedly activated when subjects engage in cognitive tasks (Cavanna, 2007). Precuneus has been associated with consciousness, and a decrease in its activity was reported in sleep, anesthesia, and vegetative states (Lückmann et al., 2014). Thalamus, the brain “relay station,” strongly connects several networks that comprise multiple cortical regions (Hwang et al., 2017). Multi-task fMRI recordings in humans suggest a robust role of the anatomical rich club as facilitating elements of functional integration in overall tasks (Shine et al., 2019). An extended analysis and discussion about the role of the rich club, in both health and disease, can be found in Griffa and Van den Heuvel (2018).

**Figure 8:**
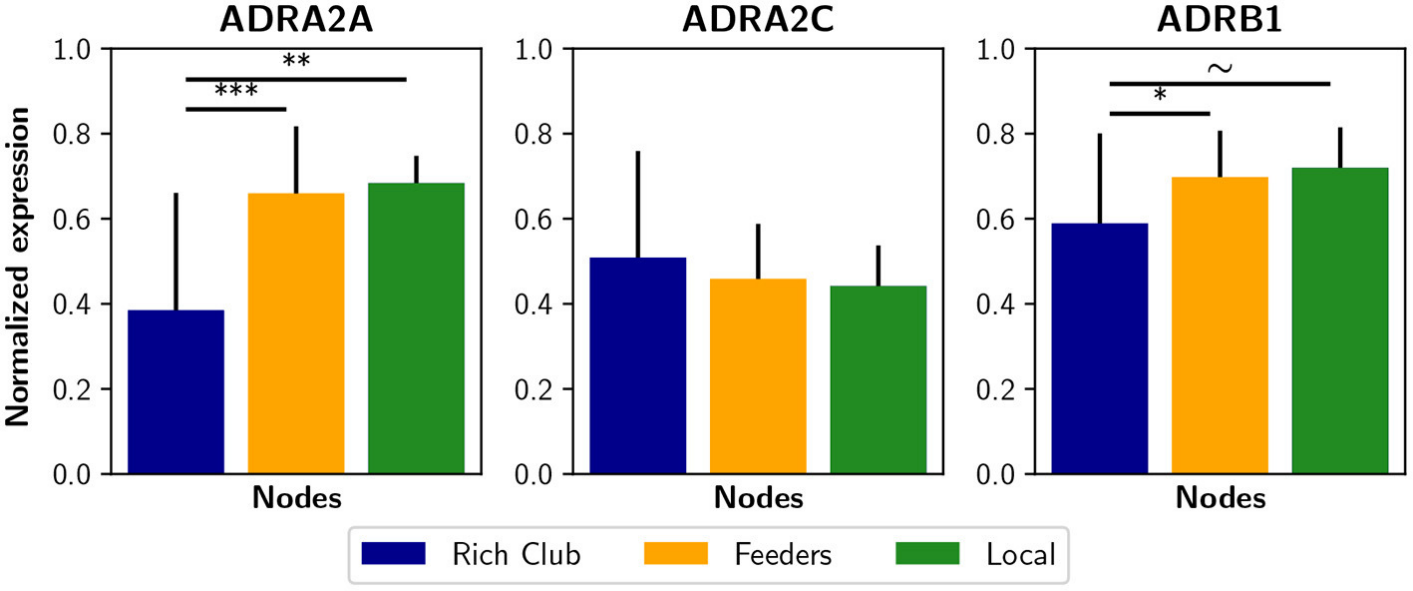
Expression of some noradrenergic receptors genes in brain regions. Genes ADRA2A, ADRA2C, and ADRAB1 are related to noradrenergic receptors α_2*A*_, α_2*C*_, and β_1_, respectively. The normalized expression was obtained from the Allen Human Brain Atlas using the AAL parcelation. Bar plots were built using the mean ± standard deviation. ***p < 0.001, **p < 0.01, *p < 0.05, ~*p* < 0.1.

The non-uniform expression of receptors across several brain areas suggests that the brain uses selective or partial neuromodulation. In this way, the effect of the noradrenergic system on filter gain may be modeled as proportional to adrenergic receptor expression. Experimentally, the optogenetic activation of the LC in mice increased average functional connectivity, which correlates with the expression of α_2_, α_1_, and β_1_ adrenergic receptors (Zerbi et al., 2019). Thus, a future research avenue in computational models may include a density-dependent noradrenergic neuromodulation with the addition of some receptors maps, obtained by positron emission tomography (PET), or even gene expression maps (Shen et al., 2012) that correlate with receptor density maps (Komorowski et al., 2017). Surprisingly, using the Allen Human Brain Atlas database (Shen et al., 2012) we found that some adrenergic receptor genes, i.e., the ADRA2A and ADRB1, are less expressed in the rich club nodes than in feeders and local nodes (Figure 8). As a consequence, the noradrenergic-mediated increase in filter gain could have a lower impact on rich club nodes. It is possible that this reduced expression constitutes a compensation for the high connectivity of rich club nodes, specially considering the higher metabolism of rich regions that exposed them to oxidative stress and neuroinflammation (Griffa and Van den Heuvel, 2018). On the other hand, receptor expression can itself be compensated by a specific sub-cellular localization or other excitability factors that may enhance the effect of noradrenaline.

It has been suggested that the effect of noradrenaline in functional connectivity is context-dependent (Shine et al., 2018b; Pfeffer et al., 2020). In that line, modeling the effect of noradrenaline in resting-state and task conditions could untangle the mechanisms behind this context-dependent effect of noradrenaline. The anatomical backbone and other dynamical parameters of this model can be substituted to study the mouse or monkey brain and to any other species for which the whole-brain white-matter connectivity is available.

Our work considers an arbitrary basal value of *r*_0_. Despite this, we reported a clear effect of the selective noradrenergic neuromodulation on functional integration, that is, some brain regions have a greater impact in the noradrenaline-mediated effect on brain function. A further improvement to our approach constitutes the use of a different benchmark, e.g., fitting the model to reproduce the empirical FC in resting-state, and then apply a homogenoeus or selective neuromodulation. Furthermore, the addition of receptors maps may be considered, as commented above.

Overall, our results offer new insights into the key regions of the human brain that, when neuromodulated via the noradrenergic system, promote transitions to integrated functional states. Our results highlight the importance of the rich club and high-strength connections in producing changes related to neuromodulation. We hope that our theoretical framework inspires new research toward clinical applications or treatments of human brain disorders caused by or associated with changes in functional and structural brain connectivity.

## 4. Methods

### 4.1. Whole-Brain Neural Mass Model

We simulated neuronal activity using the Jansen and Rit neural mass model (Jansen et al., 1993; Jansen and Rit, 1995). In this model, a brain area consists of three populations of neurons: pyramidal neurons, excitatory and inhibitory interneurons. The dynamical evolution of the three populations within the brain area is modeled by two blocks each. The first block transforms the average pulse density in average postsynaptic membrane potential (which can be either excitatory or inhibitory; Figure 1A). This block, denominated post synaptic potential (PSP), is represented by an impulse response function. For the excitatory outputs:

(1) $$\begin{array}{l} h_{E}\left(t\right)=\{\begin{array}{ll} Aate^{-at}, & t\geq 0\\ 0, & t<0 \end{array} \end{array}$$

and for the inhibitory ones

(2) $$\begin{array}{l} h_{I}\left(t\right)=\{\begin{array}{ll} Bbte^{-bt}, & t\geq 0\\ 0, & t<0, \end{array} \end{array}$$

The constants *A* and *B* define the maximum amplitude of the PSPs for the excitatory (EPSPs) and inhibitory (IPSPs) cases respectively, while *a* and *b* represent the inverse time constants for the excitatory and inhibitory postsynaptic action potentials, respectively. The second block transforms the postsynaptic membrane potential in average pulse density, and is given by a sigmoid function of the form

(3) $$\begin{array}{l} S\left(\nu ,r\right)=\frac{\zeta_{max}}{1+e^{r\left(\nu_{th}-\nu \right)}}, \end{array}$$

with ζ_*max*_ as the maximum firing rate of the neuronal population, *r* the slope of the sigmoid function, ν_*th*_ the half maximal response of the population, and ν their average PSP. Additionally, pyramidal neurons receive an external stimulus *p*(*t*), whose values are taken from a Gaussian distribution with mean μ = 2 impulses/s and standard deviation σ = 2. In this model (Figure 1A), each node *i* ∈ [1…*n*] represents a single brain area. The global coupling is scaled by a parameter α, and nodes are connected by a normalized structural connectivity matrix $\tilde{M}$ (Figure 1B). This matrix is derived from a human connectome (Deco et al., 2018) parcelated in *n* = 90 cortical and subcortical regions with the automated anatomical labeling (AAL) atlas (Tzourio-Mazoyer et al., 2002); the matrix is undirected and takes values between 0 and 1. Because long-range connections are mainly excitatory (Gilbert et al., 1990; McGuire et al., 1991), only links between the pyramidal neurons of a node *i* with pyramidal neurons of a node *j* are considered. We applied a global normalization procedure to the structural connectivity matrix *M*. The normalization consisted of dividing all the values of the matrix by the mean strength of the nodes. The resulting normalized matrix $\tilde{M}$ is defined as

(4) $$\begin{array}{l} \tilde{M}=\frac{M}{\frac{1}{n}\sum_{i=1}^{n}\sum_{j=1,j\neq i}^{n}M_{ij}} \end{array}$$

The set of equations, for a node *i*, includes the within and between nodes activity

(5) $$\begin{array}{l} {ẋ}_{0,i}\left(t\right)=y_{0,i}\left(t\right)\\ {ẏ}_{0,i}\left(t\right)=Aa\left[S\left(C_{2}x_{1,i}\left(t\right)-C_{4}x_{2,i}\left(t\right)+C\alpha z_{i}\left(t\right),r_{0}\right)\right]\\ \;-2ay_{0,i}\left(t\right)-a^{2}x_{0,i}\left(t\right)\\ {ẋ}_{1,i}\left(t\right)=y_{1,i}\left(t\right)\\ {ẏ}_{1,i}\left(t\right)=Aa\left[p\left(t\right)+S\left(C_{1}x_{0,i}\left(t\right),r_{1}\right)\right]-2ay_{1,i}\left(t\right)-a^{2}x_{1,i}\left(t\right)\\ {ẋ}_{2,i}\left(t\right)=y_{2,i}\left(t\right)\\ {ẏ}_{2,i}\left(t\right)=Bb\left[S\left(C_{3}x_{0,i}\left(t\right),r_{2}\right)\right]-2by_{2,i}\left(t\right)-b^{2}x_{2,i}\left(t\right)\\ {ẋ}_{3,i}\left(t\right)=y_{3,i}\left(t\right)\\ {ẏ}_{3,i}\left(t\right)=Aā\left[S\left(C_{2}x_{1,i}\left(t\right)-C_{4}x_{2,i}\left(t\right)+C\alpha z_{i}\left(t\right),r_{0}\right)\right]\\ \;-2āy_{3,i}\left(t\right)-{ā}^{2}x_{3,i}\left(t\right) \end{array}$$

where *x*_0_, *x*_1_, *x*_2_ correspond to the outputs of the PSP blocks of the pyramidal neurons, and excitatory and inhibitory interneurons, respectively, and *x*_3_ the long-range outputs of pyramidal neurons. The constants *C*_1_, *C*_2_, *C*_3_, and *C*_4_ scale the connectivity between the neural populations (see Figure 1A). The first pair of equations, *x*_0_ and *y*_0_, are related to the outputs of pyramidal cells to both interneurons; the second pair, *x*_1_ and *y*_1_, represent all the local excitatory inputs that the pyramidal neurons receive; *x*_2_ and *y*_2_ constitute the inhibitory contribution to pyramidal cells. An additional pair of equations (*x*_3_ and *y*_3_) are introduced to represent long-range (inter-area) connections, as they target the apical dendrites of pyramidal neurons and thus their EPSP have a larger characteristic time constant. We used the original parameter values of Jansen and Rit (Jansen et al., 1993; Jansen and Rit, 1995), except for *C*_4_: ζ_*max*_ = 5 s^−1^, ν_*th*_ = 6 mV, *r*_0_ = *r*_1_ = *r*_2_ = 0.56 mV^−1^, *a* = 100 s^−1^, *b* = 50 s^−1^, *A* = 3.25 mV, *B* = 22 mV, *C*_1_ = *C*, *C*_2_ = 0.8*C*, *C*_3_ = 0.25*C*, *C*_4_ = 0.5*C*, and *C* = 135. Changing *C*_4_ from 0.25 C to 0.5 C allowed the model to sustain oscillations in a wider range of α values. The parameters *A*, *B*, *a*, and *b* were selected to produce IPSPs longer in amplitude and latency in comparison with the EPSPs. The inverse of the characteristic time constant for the long-range EPSPs was defined as ā = 0.5*a*. This choice was based on the fact that long-range excitatory inputs of pyramidal neurons target their apical dendrites, and consequently this slows down the time course of the EPSPs at the soma (Branco and Häusser, 2011).

The input from brain areas *j* ≠ *i* to the region *i* is given by

(6) $$\begin{array}{l} z_{i}\left(t\right)=\overset{n}{\underset{j=1,j\neq i}{\sum }}{\tilde{M}}_{ij}x_{3,j}\left(t\right) \end{array}$$

The average PSP of pyramidal neurons in region *i* characterizes the EEG-like signal in the source space; it is computed as (Jansen et al., 1993; Jansen and Rit, 1995)

(7) $$\begin{array}{l} \nu {\left(t\right)}_{i}=C_{2}x_{1,i}\left(t\right)-C_{4}x_{2,i}\left(t\right)+C\alpha z_{i}\left(t\right) \end{array}$$

The firing rates of pyramidal neurons ζ_*i*_(*t*) = *S*[ν(*t*)_*i*_, *r*_0_] were used to simulate the fMRI-BOLD signals.

### 4.2. Neuromodulation

The effect of the noradrenergic system was simulated controlling the parameter *r*_0_ (filter gain; Figure 1B), which represents the sigmoid function slope of the pyramidal population, and increases the signal-to-noise ratio of pyramidal cells (Servan-Schreiber et al., 1990; Thiele and Bellgrove, 2018). Details about the relationship between the noradrenergic system and filter gain can be found in the Introduction section. Further analysis about this relationship has been presented previously in Mather et al. (2016) and Shine (2019). We analyzed the effect of the noradrenergic neuromodulation in three scenarios:

*Macro-scale:* Noradrenergic neuromodulation was studied in interaction with the cholinergic system, represented by the parameter α. The parameters were the same for all nodes. We changed the features of the connectivity matrix *M* (see Figure 2) to study the combined effect in neural coordination.

*Meso-scale:* nodes were classified in different categories, either according to the rich club organization (Van Den Heuvel and Sporns, 2011) or *s*-core decomposition of the network (see section 4.5; Garas et al., 2012; Eidsaa and Almaas, 2013). Global coupling was fixed in α = 0.65, and the basal value of *r*_0_ was 0.33 for all nodes (Figure 3). We incremented *r*_0_ in a subset of 24 nodes belonging to a particular category, and compared the results with the neuromodulation of a equal-length random subset of nodes.

Because the categories differ in the number of nodes, a fair comparison must considered subsets of equal size. To achieve that, we complemented the rich club with seven randomly selected feeder nodes, while the local nodes were complemented with 11 randomly selected feeders. Likewise, we complemented the *S*_3_ category with 14 randomly selected *S*_2_ nodes. From both the feeders and *S*_2_ nodes we selected 24 nodes randomly. All subsets consisted on 24 nodes, were generated 10 times with different random seeds and the results averaged.

*Local-scale:* Nodes were sorted using one of three metrics: node strength $K_{i}^{w}$, nodal efficiency $E_{i}^{w}$, or clustering coefficient $C_{i}^{w}$ (Rubinov and Sporns, 2010). We neuromodulated—increasing *r*_0_—node by node in increments of three, considering the metric from high to low and vice-versa (Figure 3).

### 4.3. Simulations

Following Birn et al. (2013), we ran simulations to generate the equivalent of 11 min real-time recordings, discarding the first 60 s. The system of stochastic differential equations (5) was solved with the Euler-Maruyama method, using an integration step of 1 ms. We used 10 random seeds (realizations) which controlled the initial conditions and the stochasticity of the simulations. We simulated neuronal activity sweeping the parameters α ∈ [0, 1] and *r*_0_ ∈ [0, 1], for the macro-scale scenario. In the local- and meso-scale scenarios, we swept *r*_0_ ∈ [0.33, 1] for a susbset of nodes, considering a basal value of *r*_0_ = 0.33 and a fixed α = 0.65. All the simulations were implemented in Python and the codes are freely available at: https://github.com/vandal-uv/Structural_Neuromod_2021.git. The graph analysis was performed using the Brain Connectivity Toolbox for Python (https://github.com/fiuneuro/brainconn; Rubinov and Sporns, 2010).

### 4.4. Simulated fMRI-BOLD Signals

We used the firing rates ζ_*i*_(*t*) to simulate BOLD-like signals using a generalized hemodynamic model presented in Stephan et al. (2007). In this model, an increment in the firing rate ζ_*i*_(*t*) triggers a vasodilatory response *s*_*i*_, producing blood inflow *f*_*i*_, changes in the blood volume *v*_*i*_ and deoxyhemoglobin content *q*_*i*_. The corresponding system of differential equations is

(8) $$\begin{array}{l} \dot{s_{i}}\left(t\right)=\zeta_{i}\left(t\right)-\frac{s_{i}\left(t\right)}{\tau_{s}}-\frac{f_{i}\left(t\right)-1}{\tau_{f}}\\ \dot{f_{i}}\left(t\right)=s_{i}\left(t\right)\\ \dot{v_{i}}\left(t\right)=\frac{f_{i}\left(t\right)-v_{i}{\left(t\right)}^{1/\kappa }}{\tau_{v}}\\ \dot{q_{i}}\left(t\right)=\frac{\frac{f_{i}\left(t\right)\left(1-{\left(1-E_{0}\right)}^{1/f_{i}\left(t\right)}\right)}{E_{0}}-\frac{q_{i}\left(t\right)v_{i}{\left(t\right)}^{1/\kappa }}{v_{i}\left(t\right)}}{\tau_{q}}, \end{array}$$

where τ_*s*_ = 0.65, τ_*f*_ = 0.41, τ_*v*_ = 0.98, τ_*q*_ = 0.98 represent the time constants for the signal decay, blood inflow, blood volume, and deoxyhemoglobin content, respectively. The stiffness constant (resistance of the veins to blood flow) is given by κ, and the resting-state oxygen extraction rate by *E*_0_. We used κ = 0.32 and *E*_0_ = 0.4. The BOLD-like signal of node *i*, denoted *B*_*i*_(*t*), is a non-linear function of *q*_*i*_(*t*) and *v*_*i*_(*t*)

(9) $$\begin{array}{l} B_{i}\left(t\right)=V_{0}\left[k_{1}\left(1-q_{i}\left(t\right)\right)+k_{2}\left(1-\frac{q_{i}\left(t\right)}{v_{i}\left(t\right)}\right)+k_{3}\left(1-v_{i}\left(t\right)\right)\right], \end{array}$$

where *V*_0_ = 0.04 represents the fraction of venous blood (deoxygenated) in resting-state, and *k*_1_ = 2.77, *k*_2_ = 0.2, *k*_3_ = 0.5 are kinetic constants.

The system of differential equations (8) was solved using the Euler method with an integration step of 1 ms. The signals were band-pass filtered between 0.01 and 0.1 Hz with a 3rd order Bessel filter. These BOLD-like signals were used to build the functional connectivity (FC) matrices from which the subsequent analysis of functional network properties was performed.

### 4.5. Structural Metrics

#### 4.5.1. Macro-Scale

To compare different Macro-scale features of the connectome we used four connectivity matrices (see Figure 2). The first matrix corresponds to the original human connectome matrix (Human, Figure 2A) (Deco et al., 2018). The second to a degree and strength preserving randomization of the matrix (DSPR, Figure 2B; Rubinov and Sporns, 2011). The third to a randomization, which only preserves the weight distribution of the original matrix (Random, Figure 2C). The fourth matrix was built setting to 0 all entries of *M*_*ij*_ < 0.05, and 1 otherwise (Homogeneous, Figure 2D).

#### 4.5.2. Meso-Scale

We identified the nodes belonging to the “rich club” sub-network of the graph (Van Den Heuvel and Sporns, 2011). Nodes were ranked according to degree, and then a subgraph was built using a threshold *K*, retaining the nodes with a degree greater than *K*. For each *K* value the weighted rich-club coefficient was computed as (Opsahl et al., 2008).

(10) $$\begin{array}{l} \phi^{w}\left(K\right)=\frac{W_{>K}}{\sum_{l=1}^{E_{>K}}w_{l}^{rank}} \end{array}$$

where *W*_>*K*_ is the sum of the weighted edges of the subgraph of nodes with a degree greater than *K*, *E*_>*K*_ represent the total number of edges of the subgraph, and $w_{l}^{rank}$ a vector that contains all the weighted edges of the entire network sorted from high to low values. If ϕ^*w*^(*K*) = 1, then the sum of the weights of the “rich nodes” is maximal. Otherwise, ϕ^*w*^(*K*) < 1 indicates the proportion of the weighted edges of network that are into the sub-network, and then some of the stronger connections were missed when applying the threshold *K*. The rich club coefficient was normalized in relation to DSPR surrogate graphs.

(11) $$\begin{array}{l} \phi_{norm}^{w}\left(K\right)=\frac{\phi^{w}\left(K\right)}{\phi_{rand}^{w}\left(K\right)} \end{array}$$

being $\phi_{norm}^{w}\left(K\right)$ the normalized rich club coefficient, and $\phi_{rand}^{w}\left(K\right)$ the mean rich club coefficient for a set of 1,000 random surrogates graphs. Values of $\phi_{norm}^{w}\left(K\right)>1$ indicates a rich-club organization, and nodes retained at *K* are defined as “rich club” nodes (Figure 6A). The nodes that do not belong to the rich club, but are connected with these nodes are called “feeders.” The remaining nodes correspond to “local” nodes (Figure 6B). For a maximum $\phi_{norm}^{w}\left(K\right)=1.367$ (*p* < 0.002), we identified 17 “rich club” nodes, 60 feeder nodes and 13 local nodes (Figure 6B). Because the high density of the structural matrix *M* (≈40%) hindered the discerning of the local nodes from feeders, we identify these nodes applying an absolute threshold of 0.05 to *M*. We selected this value as the maximum threshold that, when applied, preserves the fitting of the model to the empirical resting-state FC matrix.

The core-periphery organization (Hagmann et al., 2008) was analyzed performing a *s*-core decomposition (Garas et al., 2012; Eidsaa and Almaas, 2013), which identifies the cores of densely interconnected nodes in the network. The method consists in removing recursively a shell of nodes with strength less than *s* to obtain the network core nodes. The nodes were assigned to a category that corresponded to the maximal *s* value at which they are still connected to the network, defined as the critical *s*-core (Figure 7A). We defined three categories for different *s* values: *s*_1_ with 24 nodes (*s* < 1.48), *s*_2_ with 56 nodes (1.48 < *s* < 1.54), and *s*_3_ with 10 nodes (1.54 < *s* < 1.78; Figure 7B).

#### 4.5.3. Local-Scale

We employed three different metrics to characterize individual nodes. Node strength (weighted degree) was computed as

(12) $$\begin{array}{l} K_{i}^{w}=\underset{j\in N,j\neq i}{\sum }w_{ij}, \end{array}$$

where *N* is the set of nodes and *w*_*ij*_ the weighted edge of the matrix *M* (Rubinov and Sporns, 2010). We computed the nodal efficiency as

(13) $$\begin{array}{l} E_{i}^{w}=\frac{\sum_{j\in N,j\neq i}{\left(d_{ij}^{w}\right)}^{-1}}{n-1}, \end{array}$$

where $d_{ij}^{w}$ is the shortest path between the nodes *i* and *j*. Shortest paths were calculated from the sum of the inverse of the weights of *M*; the shortest path between two nodes (*i, j*) is the path that minimizes this sum (the distance). Using the shortest paths, nodal efficiency $E_{i}^{w}$ was computed. Nodes with high values of $E_{i}^{w}$ are those with high proportion of short paths to the rest of the nodes of the network (Rubinov and Sporns, 2010). Finally, we calculated the clustering coefficient for each node (Rubinov and Sporns, 2010)

(14) $$\begin{array}{l} C_{i}^{w}=\frac{2t_{i}^{w}}{k_{i}\left(k_{i}-1\right)}, \end{array}$$

where $t_{i}^{w}$ is the proportion of triangles around the node *i*, calculated as

(15) $$\begin{array}{l} t_{i}^{w}=\frac{1}{2}\underset{j,h\in N}{\sum }{\left(w_{ij}w_{ih}w_{jh}\right)}^{1/3}. \end{array}$$

A node with a high $C_{i}^{w}$ is highly connected with adjacent (local) nodes.

### 4.6. Phase Synchronization

As a measure of global synchronization, we calculated the Kuramoto order parameter *R*(*t*) (Acebrón et al., 2005) of the EEG-like signals ν(*t*) derived from the Jansen and Rit model. First, the raw signals were filtered with a 3rd order Bessel band-pass filter using their frequency of maximum power (usually between 4 and 10 Hz) ±3 Hz. Then, the instantaneous phase θ(*t*) was obtained using the Hilbert transform. The global phase synchrony is computed as:

(16) $$\begin{array}{l} \overset{-}{R}={\langle \left|{\langle e^{j\theta_{i}\left(t\right)}\rangle }_{N}\right|\rangle }_{t}, \end{array}$$

where θ_*i*_(*t*) is the phase of the oscillator *i* over time, $j=\sqrt{-1}$ the imaginary unit, |•| denotes the module, 〈〉_*N*_ denotes the average over all nodes, and 〈〉_*t*_ the average over time.

### 4.7. Functional Integration and Segregation

Functional Connectivity (FC) matrices were built from Pearson correlations of the entire BOLD-like time series. Instead of employing an absolute or proportional thresholding, we thresholded the FC matrices using Fourier transform (FT) surrogate data (Lancaster et al., 2018) to avoid the problem of introducing spurious correlations (Fornito et al., 2013). The FT algorithm uses a phase randomization process to destroy pairwise correlations, preserving the spectral properties of the signals (the surrogates have the same power spectrum as the original data). We generated 500 surrogate time series of the original set of BOLD-like signals, to obtain the surrogate sFCs matrices. For each one of the (*n*^2^ − *n*)/2 possible connectivity pairs (with *n* = 90) we fitted a normal distribution of the surrogate values. Using these distributions, we tested the hypothesis that a pairwise correlation is higher than chance (that is, the value is at the right of the surrogate distribution).

To reject the null hypothesis, we selected a *p*-value equal to 0.05, and corrected for multiple comparisons with the FDR Benjamini-Hochberg procedure (Benjamini and Hochberg, 1995) to decrease the probability of making type I errors (false positives). The entries of the sFC matrix associated with a *p* > 0.05 were set to 0. The result is a thresholded, undirected, and weighted (with only positive values) sFC matrix.

Integration was evaluated over the thresholded FC matrices. We employed the weighted version of the global efficiency (Latora and Marchiori, 2001). This measure of integration is based on paths over the graph: it is defined as the inverse of the average shortest path length. This metric is computed as

(17) $$\begin{array}{l} E^{w}=\frac{1}{n}\underset{i\in N}{\sum }\frac{\sum_{j\in N,j\neq i}{\left(d_{ij}^{w}\right)}^{-1}}{n-1}, \end{array}$$

being *N* the set of all nodes, *n* number of nodes, and $d_{ij}^{w}$ the shortest path between the nodes *i* and *j*.

Segregation was quantified using modularity *Q*^*w*^, a metric for the detection of the network's communities (Rubinov and Sporns, 2010). The detection of so-called communities or network modules in the thresholded FC matrix, was based on the Louvain's algorithm (Newman, 2006; Blondel et al., 2008). We used the weighted version of the modularity (Newman, 2004) defined as

(18) $$\begin{array}{l} Q^{w}=\frac{1}{l^{w}}\underset{i,j\in N}{\sum }\left[w_{ij}-\frac{k_{i}^{w}k_{j}^{w}}{l^{w}}\right]\delta_{m_{i},m_{j}} \end{array}$$

where *w*_*ij*_ is the weight of the link between the nodes *i* and *j*, *l*^*w*^ is the total number of weighted links of the network, *m*_*i*_ (*m*_*j*_) the module of the node *i* (*j*), and $k_{i}^{w}$ (*m*_*j*_) the weighted degree (named also strength) of *i* (*j*). The algorithm assigns a module to each node in a way that maximizes the modularity (18). The Kronecker delta δ_*m*_*i*_,*m*_*j*__ is equal to 1 when *m*_*i*_ = *m*_*j*_ (that is, when two nodes belongs to the same module), and 0 otherwise.

Because the Louvain's algorithm is stochastic, we employed the consensus-clustering algorithm (Lancichinetti and Fortunato, 2012). We ran the Louvain's algorithm 200 times with the resolution parameter set to 1.0 (this parameter controls the size of the detected modules; larger values of this parameter allows the detection of smaller modules). Then, we built an agreement matrix *G*, whose entries *G*_*ij*_ ∈ [0, 1] indicates the proportion of partitions in which the pairs of nodes (*i, j*) share the same module. Then, we applied an absolute threshold of 0.5 to the matrix *G*, and ran the Louvain's algorithm again 200 times using *G* as input, producing a new consensus matrix *G*′. This last step was repeated until convergence to a unique partition.

### 4.8. Functional Connectivity Dynamics

The FCD matrix captures the evolution of FC patterns and, consequently, the dynamical richness of the network (Hansen et al., 2015; Cabral et al., 2017). We used the sliding window approach (Hansen et al., 2015; Orio et al., 2018), with windows of 100 s length and a displacement of 2 s between consecutive windows. The length was chosen on the basis of the lower limit of the band-pass filter (0.01 Hz), in order to minimize spurious correlations (Leonardi and Van De Ville, 2015). For each window, a FC matrix was calculated from the Pearson correlation of BOLD-like signals. We obtained 251 weighted and undirected FC matrices from the 600 s simulated BOLD-like signals. The upper triangular of each FC matrix is unfolded to make a vector, and the FCD is built by calculating the Clarkson distance $\lambda \left(x,y\right)=\frac{1}{\sqrt{2}}\|\frac{x}{||x||}-\frac{y}{||y||}\|$ between each pair of FCs (Clarkson, 1936).

(19) $$\begin{array}{l} FCD_{ij}=\lambda \left(FC\left(t_{i}\right),FC\left(t_{j}\right)\right) \end{array}$$

### 4.9. Gene Expression Maps

To quantify the expression of some noradrenergic receptor genes in brain regions, we used the microarray expression data of the Allen Human Brain Atlas (Shen et al., 2012). The dataset was processed and normalized employing the Abagen library for Python (https://github.com/rmarkello/abagen/tree/0.1; Arnatkevicute et al., 2019), and then parcellated using the AAL atlas (Tzourio-Mazoyer et al., 2002). We compared the expression of the ADRA2A, ADRA2C, and ADRB1 genes in rich club, feeders and local nodes. Statistical comparison was performed with a Student's *t*-test for independent samples.

## Data Availability Statement

The datasets presented in this study can be found in online repositories. The names of the repository/repositories and accession number(s) can be found at: https://github.com/vandal-uv/Structural_Neuromod_2021.

## Author Contributions

CC-O, SC, RC, and PO contributed to conception and design of the study and wrote the manuscript. CC-O performed the simulations and statistical analysis. All authors contributed to manuscript revision, read, and approved the submitted version.

## Conflict of Interest

The authors declare that the research was conducted in the absence of any commercial or financial relationships that could be construed as a potential conflict of interest.

## References

[B1] Acebrón J. A. Bonilla L. L. Vicente C. J. P. Ritort F. Spigler R. (2005). The kuramoto model: a simple paradigm for synchronization phenomena. Rev. Modern Phys. 77:137. 10.1103/RevModPhys.77.137

[B2] Allen E. A. Damaraju E. Plis S. M. Erhardt E. B. Eichele T. Calhoun V. D. (2014). Tracking whole-brain connectivity dynamics in the resting state. Cereb. Cortex 24, 663–676. 10.1093/cercor/bhs352 23146964 PMC3920766

[B3] Arnatkevicute A. Fulcher B. D. Fornito A. (2019). A practical guide to linking brain-wide gene expression and neuroimaging data. Neuroimage 189, 353–367. 10.1016/j.neuroimage.2019.01.011 30648605

[B4] Aston-Jones G. Cohen J. D. (2005). An integrative theory of locus coeruleus-norepinephrine function: adaptive gain and optimal performance. Annu. Rev. Neurosci. 28, 403–450. 10.1146/annurev.neuro.28.061604.135709 16022602

[B5] Beliveau V. Ganz M. Feng L. Ozenne B. Højgaard L. Fisher P. M.. (2017). A high-resolution *in vivo* atlas of the human brain's serotonin system. J. Neurosci. 37, 120–128. 10.1523/JNEUROSCI.2830-16.2016 28053035 PMC5214625

[B6] Benjamini Y. Hochberg Y. (1995). Controlling the false discovery rate: a practical and powerful approach to multiple testing. J. R. Stat. Soc. Ser. B 57, 289–300. 10.1111/j.2517-6161.1995.tb02031.x

[B7] Birn R. M. Molloy E. K. Patriat R. Parker T. Meier T. B. Kirk G. R.. (2013). The effect of scan length on the reliability of resting-state fMRI connectivity estimates. Neuroimage 83, 550–558. 10.1016/j.neuroimage.2013.05.099 23747458 PMC4104183

[B8] Blondel V. D. Guillaume J.-L. Lambiotte R. Lefebvre E. (2008). Fast unfolding of communities in large networks. J. Stat. Mech. 2008:P10008. 10.1088/1742-5468/2008/10/P10008

[B9] Branco T. Häusser M. (2011). Synaptic integration gradients in single cortical pyramidal cell dendrites. Neuron 69, 885–892. 10.1016/j.neuron.2011.02.006 21382549 PMC6420135

[B10] Bullmore E. Sporns O. (2009). Complex brain networks: graph theoretical analysis of structural and functional systems. Nat. Rev. Neurosci. 10, 186–198. 10.1038/nrn2575 19190637

[B11] Cabral J. Kringelbach M. L. Deco G. (2017). Functional connectivity dynamically evolves on multiple time-scales over a static structural connectome: models and mechanisms. NeuroImage 160, 84–96. 10.1016/j.neuroimage.2017.03.045 28343985

[B12] Cabral J. Luckhoo H. Woolrich M. Joensson M. Mohseni H. Baker A.. (2014). Exploring mechanisms of spontaneous functional connectivity in MEG: how delayed network interactions lead to structured amplitude envelopes of band-pass filtered oscillations. Neuroimage 90, 423–435. 10.1016/j.neuroimage.2013.11.047 24321555

[B13] Castro S. El-Deredy W. Battaglia D. Orio P. (2020). Cortical ignition dynamics is tightly linked to the core organisation of the human connectome. PLoS Comput. Biol. 16:e1007686. 10.1371/journal.pcbi.1007686 32735580 PMC7423150

[B14] Cavanna A. E. (2007). The precuneus and consciousness. CNS Spectr. 12, 545–552. 10.1017/S1092852900021295 17603406

[B15] Clarkson J. A. (1936). Uniformly convex spaces. Trans. Am. Math. Soc. 40, 396–414. 10.1090/S0002-9947-1936-1501880-4

[B16] Cohen J. R. D'Esposito M. (2016). The segregation and integration of distinct brain networks and their relationship to cognition. J. Neurosci. 36, 12083–12094. 10.1523/JNEUROSCI.2965-15.2016 27903719 PMC5148214

[B17] Coronel-Oliveros C. Cofré R. Orio P. (2021). Cholinergic neuromodulation of inhibitory interneurons facilitates functional integration in whole-brain models. PLoS Comput. Biol. 17:e1008737. 10.1371/journal.pcbi.1008737 33600402 PMC7924765

[B18] Deco G. Cruzat J. Cabral J. Knudsen G. M. Carhart-Harris R. L. Whybrow P. C.. (2018). Whole-brain multimodal neuroimaging model using serotonin receptor maps explains non-linear functional effects of LSD. Curr. Biol. 28, 3065–3074. 10.1016/j.cub.2018.07.083 30270185

[B19] Deco G. Jirsa V. K. (2012). Ongoing cortical activity at rest: criticality, multistability, and ghost attractors. J. neurosci. 32, 3366–75. 10.1523/JNEUROSCI.2523-11.2012 22399758 PMC6621046

[B20] Deco G. Van Hartevelt T. J. Fernandes H. M. Stevner A. Kringelbach M. L. (2017). The most relevant human brain regions for functional connectivity: evidence for a dynamical workspace of binding nodes from whole-brain computational modelling. Neuroimage 146, 197–210. 10.1016/j.neuroimage.2016.10.047 27825955

[B21] Dehaene S. Changeux J. P. (2011). Experimental and theoretical approaches to conscious processing. Neuron 70, 200–227. 10.1016/j.neuron.2011.03.018 21521609

[B22] Eidsaa M. Almaas E. (2013). S-core network decomposition: a generalization of k-core analysis to weighted networks. Phys. Rev. E 88:062819. 10.1103/PhysRevE.88.062819 24483523

[B23] Fornito A. Zalesky A. Breakspear M. (2013). Graph analysis of the human connectome: promise, progress, and pitfalls. Neuroimage 80, 426–444. 10.1016/j.neuroimage.2013.04.087 23643999

[B24] Foster B. L. He B. J. Honey C. J. Jerbi K. Maier A. Saalmann Y. B. (2016). Spontaneous neural dynamics and multi-scale network organization. Front. Syst. Neurosci. 10:7. 10.3389/fnsys.2016.00007 26903823 PMC4746329

[B25] Fukushima M. Sporns O. (2020). Structural determinants of dynamic fluctuations between segregation and integration on the human connectome. Commun. Biol. 3, 1–11. 10.1038/s42003-020-01331-3 33097809 PMC7584581

[B26] Fuxe K. Dahlström A. B. Jonsson G. Marcellino D. Guescini M. Dam M.. (2010). The discovery of central monoamine neurons gave volume transmission to the wired brain. Prog. Neurobiol. 90, 82–100. 10.1016/j.pneurobio.2009.10.012 19853007

[B27] Garas A. Schweitzer F. Havlin S. (2012). A k-shell decomposition method for weighted networks. New J. Phys. 14:083030. 10.1088/1367-2630/14/8/083030

[B28] Gilbert C. D. Hirsch J. A. Wiesel T. N. (1990). Lateral interactions in visual cortex. Cold Spring Harb. Symp. Quant. Biol. 55, 663–677. 10.1101/SQB.1990.055.01.063 2132846

[B29] González G. F. Van der Molen M. Žarić G. Bonte M. Tijms J. Blomert L.. (2016). Graph analysis of EEG resting state functional networks in dyslexic readers. Clin. Neurophysiol. 127, 3165–3175. 10.1016/j.clinph.2016.06.023 27476025

[B30] Griffa A. Van den Heuvel M. P. (2018). Rich-club neurocircuitry: function, evolution, and vulnerability. Dial. Clin. Neurosci. 20, 121–132. 10.31887/DCNS.2018.20.2/agriffa 30250389 PMC6136122

[B31] Guan S. Jiang R. Bian H. Yuan J. Xu P. Meng C.. (2020). The profiles of non-stationarity and non-linearity in the time series of resting-state brain networks. Front. Neurosci. 14:493. 10.3389/fnins.2020.00493 32595440 PMC7300942

[B32] Hagmann P. Cammoun L. Gigandet X. Meuli R. Honey C. J. Wedeen V. J.. (2008). Mapping the structural core of human cerebral cortex. PLoS Biol. 6:e159. 10.1371/journal.pbio.0060159 18597554 PMC2443193

[B33] Hansen E. C. Battaglia D. Spiegler A. Deco G. Jirsa V. K. (2015). Functional connectivity dynamics: modeling the switching behavior of the resting state. Neuroimage 105, 525–535. 10.1016/j.neuroimage.2014.11.001 25462790

[B34] Herzog R. Mediano P. A. Rosas F. E. Carhart-Harris R. Perl Y. S. Tagliazucchi E.. (2020). A mechanistic model of the neural entropy increase elicited by psychedelic drugs. Sci. Rep. 10, 1–12. 10.1038/s41598-020-74060-6 33082424 PMC7575594

[B35] Hwang K. Bertolero M. A. Liu W. B. D'esposito M. (2017). The human thalamus is an integrative hub for functional brain networks. J. Neurosci. 37, 5594–5607. 10.1523/JNEUROSCI.0067-17.2017 28450543 PMC5469300

[B36] Jansen B. H. Rit V. G. (1995). Electroencephalogram and visual evoked potential generation in a mathematical model of coupled cortical columns. Biol. Cybern. 73, 357–366. 10.1007/BF00199471 7578475

[B37] Jansen B. H. Zouridakis G. Brandt M. E. (1993). A neurophysiologically-based mathematical model of flash visual evoked potentials. Biol. Cybern. 68, 275–283. 10.1007/BF00224863 8452897

[B38] Kelso J. S. (2012). Multistability and metastability: understanding dynamic coordination in the brain. Philos. Trans. R. Soc. B Biol. Sci. 367, 906–918. 10.1098/rstb.2011.0351 22371613 PMC3282307

[B39] Komorowski A. James G. Philippe C. Gryglewski G. Bauer A. Hienert M.. (2017). Association of protein distribution and gene expression revealed by pet and post-mortem quantification in the serotonergic system of the human brain. Cereb. Cortex 27, 117–130. 10.1093/cercor/bhw355 27909009 PMC5939202

[B40] Kringelbach M. L. Cruzat J. Cabral J. Knudsen G. M. Carhart-Harris R. Whybrow P. C.. (2020). Dynamic coupling of whole-brain neuronal and neurotransmitter systems. Proc. Natl. Acad. Sci. U.S.A. 117, 9566–9576. 10.1073/pnas.1921475117 32284420 PMC7196827

[B41] Lancaster G. Iatsenko D. Pidde A. Ticcinelli V. Stefanovska A. (2018). Surrogate data for hypothesis testing of physical systems. Phys. Rep. 748, 1–60. 10.1016/j.physrep.2018.06.001

[B42] Lancichinetti A. Fortunato S. (2012). Consensus clustering in complex networks. Sci. Rep. 2:336. 10.1038/srep00336 22468223 PMC3313482

[B43] Latora V. Marchiori M. (2001). Efficient behavior of small-world networks. Phys. Rev. Lett. 87:198701. 10.1103/PhysRevLett.87.198701 11690461

[B44] Lee T.-H. Greening S. G. Ueno T. Clewett D. Ponzio A. Sakaki M.. (2018). Arousal increases neural gain via the locus coeruleus-noradrenaline system in younger adults but not in older adults. Nat. Hum. Behav. 2, 356–366. 10.1038/s41562-018-0344-1 30320223 PMC6176734

[B45] Leonardi N. Van De Ville D. (2015). On spurious and real fluctuations of dynamic functional connectivity during rest. Neuroimage 104, 430–436. 10.1016/j.neuroimage.2014.09.007 25234118

[B46] Lord L.-D. Stevner A. B. Deco G. Kringelbach M. L. (2017). Understanding principles of integration and segregation using whole-brain computational connectomics: implications for neuropsychiatric disorders. Philos. Trans. R. Soc. A 375:20160283. 10.1098/rsta.2016.0283 28507228 PMC5434074

[B47] Lückmann H. C. Jacobs H. I. Sack A. T. (2014). The cross-functional role of frontoparietal regions in cognition: internal attention as the overarching mechanism. Prog. Neurobiol. 116, 66–86. 10.1016/j.pneurobio.2014.02.002 24530293

[B48] Mather M. Clewett D. Sakaki M. Harley C. W. (2016). Norepinephrine ignites local hotspots of neuronal excitation: how arousal amplifies selectivity in perception and memory. Behav. Brain Sci. 39:e200. 10.1017/S0140525X15000667 26126507 PMC5830137

[B49] McGuire B. A. Gilbert C. D. Rivlin P. K. Wiesel T. N. (1991). Targets of horizontal connections in macaque primary visual cortex. J. Compar. Neurol. 305, 370–392. 10.1002/cne.903050303 1709953

[B50] Miron-Shahar Y. Kantelhardt J. W. Grinberg A. Hassin-Baer S. Blatt I. Inzelberg R.. (2019). Excessive phase synchronization in cortical activation during locomotion in persons with Parkinson's disease. Parkinsonism Relat. Disord. 65, 210–216. 10.1016/j.parkreldis.2019.05.030 31383631

[B51] Newman M. E. (2004). Analysis of weighted networks. Phys. Rev. E 70:056131. 10.1103/PhysRevE.70.056131 15600716

[B52] Newman M. E. (2006). Modularity and community structure in networks. Proc. Natl. Acad. Sci. U.S.A. 103, 8577–8582. 10.1073/pnas.0601602103 16723398 PMC1482622

[B53] Opsahl T. Colizza V. Panzarasa P. Ramasco J. J. (2008). Prominence and control: the weighted rich-club effect. Phys. Rev. Lett. 101:168702. 10.1103/PhysRevLett.101.168702 18999722

[B54] Orio P. Gatica M. Herzog R. Maidana J. P. Castro S. Xu K. (2018). Chaos versus noise as drivers of multistability in neural networks. Chaos 28:106321. 10.1063/1.5043447 30384618

[B55] Pfeffer T. Ponce-Alvarez A. Meindertsma T. Gahnström C. van den Brink R. L. Nolte G.. (2020). Circuit mechanisms for chemical modulation of cortex-wide network interactions and exploration behavior. bioRxiv. 10.1101/2020.06.25.171199 PMC8284895 34272245

[B56] Reimer J. McGinley M. J. Liu Y. Rodenkirch C. Wang Q. McCormick D. A.. (2016). Pupil fluctuations track rapid changes in adrenergic and cholinergic activity in cortex. Nat. Commun. 7, 1–7. 10.1038/ncomms13289 27824036 PMC5105162

[B57] Rubinov M. Sporns O. (2010). Complex network measures of brain connectivity: uses and interpretations. Neuroimage 52, 1059–1069. 10.1016/j.neuroimage.2009.10.003 19819337

[B58] Rubinov M. Sporns O. (2011). Weight-conserving characterization of complex functional brain networks. Neuroimage 56, 2068–2079. 10.1016/j.neuroimage.2011.03.069 21459148

[B59] Servan-Schreiber D. Printz H. Cohen J. D. (1990). A network model of catecholamine effects: gain, signal-to-noise ratio, and behavior. Science 249, 892–895. 10.1126/science.2392679 2392679

[B60] Shen E. H. Overly C. C. Jones A. R. (2012). The allen human brain atlas: comprehensive gene expression mapping of the human brain. Trends Neurosci. 35, 711–714. 10.1016/j.tins.2012.09.005 23041053

[B61] Shine J. M. (2019). Neuromodulatory influences on integration and segregation in the brain. Trends Cogn. Sci. 23, 572–583. 10.1016/j.tics.2019.04.002 31076192

[B62] Shine J. M. Aburn M. J. Breakspear M. Poldrack R. A. (2018a). The modulation of neural gain facilitates a transition between functional segregation and integration in the brain. Elife 7:e31130. 10.7554/eLife.31130 29376825 PMC5818252

[B63] Shine J. M. Bissett P. G. Bell P. T. Koyejo O. Balsters J. H. Gorgolewski K. J.. (2016). The dynamics of functional brain networks: integrated network states during cognitive task performance. Neuron 92, 544–554. 10.1016/j.neuron.2016.09.018 27693256 PMC5073034

[B64] Shine J. M. Breakspear M. Bell P. T. Martens K. A. E. Shine R. Koyejo O.. (2019). Human cognition involves the dynamic integration of neural activity and neuromodulatory systems. Nat. Neurosci. 22, 289–296. 10.1038/s41593-018-0312-0 30664771

[B65] Shine J. M. van den Brink R. L. Hernaus D. Nieuwenhuis S. Poldrack R. A. (2018b). Catecholaminergic manipulation alters dynamic network topology across cognitive states. Netw. Neurosci. 2, 381–396. 10.1162/netn_a_00042 30294705 PMC6145851

[B66] Sporns O. (2013). Network attributes for segregation and integration in the human brain. Curr. Opin. Neurobiol. 23, 162–171. 10.1016/j.conb.2012.11.015 23294553

[B67] Stephan K. E. Weiskopf N. Drysdale P. M. Robinson P. A. Friston K. J. (2007). Comparing hemodynamic models with DCM. Neuroimage 38, 387–401. 10.1016/j.neuroimage.2007.07.040 17884583 PMC2636182

[B68] Thiele A. Bellgrove M. A. (2018). Neuromodulation of attention. Neuron 97, 769–785. 10.1016/j.neuron.2018.01.008 29470969 PMC6204752

[B69] Tognoli E. Kelso J. S. (2014). The metastable brain. Neuron 81, 35–48. 10.1016/j.neuron.2013.12.022 24411730 PMC3997258

[B70] Tononi G. (2004). An information integration theory of consciousness. BMC Neurosci. 5:42. 10.1186/1471-2202-5-42 15522121 PMC543470

[B71] Tzourio-Mazoyer N. Landeau B. Papathanassiou D. Crivello F. Etard O. Delcroix N.. (2002). Automated anatomical labeling of activations in SPM using a macroscopic anatomical parcellation of the MNI MRI single-subject brain. Neuroimage 15, 273–289. 10.1006/nimg.2001.0978 11771995

[B72] Van Den Heuvel M. P. Sporns O. (2011). Rich-club organization of the human connectome. J. Neurosci. 31, 15775–15786. 10.1523/JNEUROSCI.3539-11.2011 22049421 PMC6623027

[B73] Wang R. Lin P. Liu M. Wu Y. Zhou T. Zhou C. (2019). Hierarchical connectome modes and critical state jointly maximize human brain functional diversity. Phys. Rev. Lett. 123:038301. 10.1103/PhysRevLett.123.038301 31386449

[B74] Wang R. Liu M. Cheng X. Wu Y. Hildebrandt A. Zhou C. (2021). Segregation, integration and balance of large-scale resting brain networks configure different cognitive abilities. arXiv preprint arXiv:2103.00475. 10.1073/pnas.2022288118 34074762 PMC8201916

[B75] Xia M. Wang J. He Y. (2013). Brainnet viewer: a network visualization tool for human brain connectomics. PLoS ONE 8:e68910. 10.1371/journal.pone.0068910 23861951 PMC3701683

[B76] Zamora-López G. Chen Y. Deco G. Kringelbach M. L. Zhou C. (2016). Functional complexity emerging from anatomical constraints in the brain: the significance of network modularity and rich-clubs. Sci. Rep. 6:38424. 10.1038/srep38424 27917958 PMC5137167

[B77] Zerbi V. Floriou-Servou A. Markicevic M. Vermeiren Y. Sturman O. Privitera M.. (2019). Rapid reconfiguration of the functional connectome after chemogenetic locus coeruleus activation. Neuron 103, 702–718. 10.1016/j.neuron.2019.05.034 31227310

